# Selective inhibition of carbonic anhydrase IX by sulphonylated 1,2,3-triazole incorporated benzenesulphonamides capable of inducing apoptosis

**DOI:** 10.1080/14756366.2022.2077333

**Published:** 2022-05-26

**Authors:** Kiran Siwach, Amit Kumar, Harish Panchal, Rajiv Kumar, Jitender Kumar Bhardwaj, Andrea Angeli, Claudiu T. Supuran, Pawan K. Sharma

**Affiliations:** a Department of Chemistry, Kurukshetra University, Kurukshetra, India; b Reproductive Physiology Laboratory, Department of Zoology, Kurukshetra University, Kurukshetra, India; c Department of Chemistry, Ch. Mani Ram Godara Government College for Women, Bhodia Khera, Fatehabad, India; d Department of Neurosciences, Psychology, Drug Research and Child Health, Pharmaceutical and Nutraceutical Section, University of Florence, Florence, Italy

**Keywords:** Carbonic anhydrase inhibitors, hCA isoforms, benzenesulphonamide, triazole, apoptosis

## Abstract

In search of selective carbonic anhydrase (CA) IX inhibitors endowed with apoptotic inducing properties, we designed and synthesised two subsets of 4- and 3-(5-aryl-(4-phenylsulphonyl)-1*H*-1,2,3-triazol-1-yl)benzenesulphonamides. All compounds were assayed for human carbonic anhydrase (hCA) isoforms I, II, IV, and IX inhibition. Isoforms hCA I and hCA IV were weakly inhibited by most of the synthesised compounds. Many four-substituted benzenesulphonamides displayed low nanomolar inhibition against isoform hCA II, unlike the three-substituted analogues. All target compounds exhibited good inhibition profile with K_I_ values ranging from 16.4 to 66.0 nM against tumour-associated isoform hCA IX. Some selective and potent inhibitors of hCA IX were assayed for *in vitro* apoptotic induction in goat testicular cells. Compounds **10d** and **10h** showed interesting apoptotic induction potential. The present study may provide insights into a strategy for the design of novel anticancer agents based on hCA inhibitors endowed with apoptotic interference.

## 1. Introduction

Irrespective of the therapeutic developments, cancer is the second leading cause of premature death worldwide.^1^^,^^2^ The hypoxic microenvironment of tumour cells is the hallmark for many types of cancer and was described as the “Warburg effect”.^3^ The transcription factors hypoxia-inducible factor 1 and 2 (HIF-1/2) sense oxygen levels of tumour cells and regulate various genes involved in metabolism, pH balance, and angiogenesis.^4^ Several carbonic anhydrase (CA) isoforms, specifically hCA (human carbonic anhydrase) IX and XII exhibit active participation in these hypoxic tumours related metabolic processes and have been validated as antimetastatic or anti-tumour drug targets.^4^^,^^5^ Carbonic anhydrases (CAs, EC 4.2.1.1) are a superfamily of metalloenzymes widespread in most living organisms and these catalyse a very essential physiological reaction, the reversible hydration of CO_2_ generating bicarbonate ions (HCO_3_^−^) and protons (H^+^), in addition to the catalysis of a variety of other physiological reactions.^6^^,^^7^ Till date, 16 different α-CA isoforms have been identified in mammals. The active site of all the catalytically active α-CAs consists of Zn (II) ion co-ordinated with three histidine residues and a water molecule or hydroxide ion. In humans, there are fifteen different isoforms, out of which the catalytically active isoforms are involved in vital physiological processes associated with respiration, transport of CO_2_ between metabolising tissues and lungs, pH regulation, electrolyte secretion in variety of tissues and organs, biosynthetic reactions, bone resorption, calcification, tumourigenicity, and many other physiological as well as pathologic processes.^8^^,^^9^ hCA I and II are ubiquitous isoforms which may serve as targets for some diseases and off-targets for others.^10^ hCA IV, together with hCA II and XII, has been proved to be a good target for antiglaucoma drugs and is also involved in retinitis pigmentosa and stroke.^10^^,^^11^ hCA IX has been described as the tumour-associated isozyme due to its limited presence in normal tissues, overexpression in solid tumours and active contribution in survival, migration and invasion of tumour cells by regulating extracellular pH in tumour microenvironment.^4^^,^^12^ HIF-1 pathway mediates the overexpression of hCA IX in hypoxic tumour microenvironment.^5^^,^^13^ hCA IX has been considered as a valuable marker for cancer, and the development of hCA IX inhibitors with selectivity over widely distributed isoforms hCA I/II is a potential strategy for designing anticancer agents. Another hallmark found to be associated with cancer is the inhibition or delay of apoptosis and this condition leads to the increased invasiveness during tumour progression, angiogenesis stimulation, deregulated cell proliferation, and interference with cell differentiation. Apoptosis is a highly regulated natural mechanism for programmed cell death which is controlled by various intracellular as well as extracellular signalling pathways. Among non-surgical treatments of cancer, targeting apoptosis is one of the highly successful approaches as it is non-specific to the type of cancer.^14^^,^^15^

**Figure 1.**
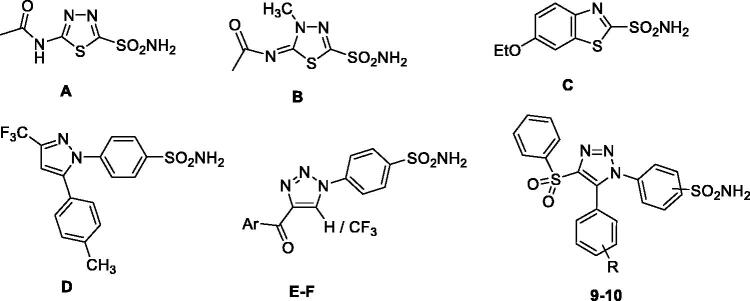
Clinically used drugs as hCA inhibitors **(**A) acetazolamide, (B) methazolamide, (C) ethoxzolamide, (D) celecoxib;^7^ 1,2,3-triazole based reported hCA inhibitors (E–F)^16^ along with newly designed and synthesised target compounds **9–10**.

In view of the obvious advantage of combining the isoform-selective hCA inhibition and apoptosis by a single chemical agent, it is desirable to develop potent and selective inhibitors of tumour-associated isoform hCA IX endowed with apoptosis induction properties. Sulphonamide derivatives have been extensively explored for CA inhibition activities in search of selective hCA IX inhibitors. A number of sulphonamide bearing clinically used drugs have been reported to possess significant CA inhibitory properties including acetazolamide (**A**), methazolamide (**B**), ethoxzolamide (**C**), celecoxib (**D**), etc. (Figure 1) which are in use for many years as diuretics, antiglaucoma agents, and antiepileptics. 1,2,3-triazoles and their derivatives have attained significant attention over past few years due to their wide spectrum of biological activities.^17^ Our research group has explored 1,2,3-triazole scaffold in search of effective inhibitors of various hCA isoforms.^11^^,^^16^^,^^18–20^ The interesting outcomes of these studies, coupled with the fact that the data on the apoptotic induction properties of selective hCA inhibitors is rare, prompted us to explore the biological assessment of novel compounds **9–10** of this versatile scaffold as inhibitors of hCA I, II, IV, and IX isoforms capable of inducing apoptosis.

## 2. Materials and methods

### 2.1. Chemistry

#### 2.1.1. General

All reagents and solvents obtained from commercial sources were used without further purification. The air- or moisture-sensitive reactions were carried out under nitrogen atmosphere using dry glassware. All the reactions were monitored by thin-layer chromatography (TLC) on TLC silica gel on F_254_ aluminium plates using appropriate solvents or mixture of solvents as eluent while UV lamp was used to visualise the spots. Melting points were determined on POPULAR digital melting point apparatus and were uncorrected. IR spectra were recorded on ABB MB 3000 FT-IR spectrometer. The high-resolution mass spectrometry (HRMS) analysis was performed with a SCIEX Triple TOF 5600 spectrometer using acetonitrile as solvent. ^1^H and ^13 ^C NMR spectra were recorded on Bruker Avance III at 400 MHz and 100 MHz, respectively. Deuterated dimethyl sulphoxide (DMSO-d_6_) was used as solvent, and tetramethylsilane (TMS) was used as internal standard for obtaining NMR spectra at room temperature. All chemical shifts are reported as δ values in parts per million (ppm) with reference to TMS. Multiplicities are abbreviated as singlet (s), doublet (d), doublet of doublets (dd), doublet of doublets of doublets (ddd), doublet of triplets (dt), triplet (t), triplet of triplets (tt), and multiplet (m) for NMR assignment. Strong (s) and medium (m) are used for peak intensities in IR assignments.

#### 2.1.2. Synthesis of 4- and 3-(5-aryl-(4-phenylsulphonyl)-1H-1,2,3-triazol-1-yl)benzenesulphonamide (9a–9k) and (10a–10k)

General procedure: The appropriate β-ketosulphone **4a–4k** (0.6 mmol) was dissolved in 3 ml DMSO, followed by the addition of piperidine (5 mol%) as catalyst. Then, appropriate azidobenzenesulphonamide **7–8** (0.66 mmol) was added, and the reaction mixture was stirred at room temperature for 24 h. The progress of reaction was monitored through TLC using mixture of methanol and chloroform as developing solvent. The reaction was quenched with water (5 ml) to yield precipitates which were filtered, washed several times with water, and then dried to afford pure solid compound which was recrystallized using ethanol.

##### 2.1.2.1. 4-(5-phenyl-4-(phenylsulphonyl)-1H-1,2,3-triazol-1-yl)benzenesulphonamide (9a)

Yield 98%; white solid; mp: 214–216 °C; IR (ν, cm^−1^): 3310, 3225 (m, N–H stretch), 1335, 1149 (s, S=O stretch); ^1^H NMR (400 MHz, DMSO-d_6_) δ (ppm): 7.87–7.82 (m, 4H, Ar), 7.76 (tt, *J =* 7.4 Hz, *J =* 1.2 Hz, 1H, Ar), 7.66–7.62 (m, 4H, Ar), 7.53–7.39 (m, 7H, Ar, SO_2_NH_2_); ^13 ^C NMR (100 MHz, DMSO-d_6_) δ (ppm): 145.78, 145.22, 140.61, 140.21, 137.79, 134.80, 130.97, 130.13, 128.85, 128.00, 127.22, 127.17, 124.30; HRMS (ESI-MS) *m/z* 441.0723 (M + H)^+^, C_20_H_16_N_4_O_4_S_2_H^+^, calcd 441.0691.

##### 2.1.2.2. 4-(4-(phenylsulphonyl)-5-(p-tolyl)-1H-1,2,3-triazol-1-yl)benzenesulphonamide (9b)

Yield 87%; off-white solid; mp: 205–207 °C; IR (ν, cm^−1^): 3348, 3240 (m, N–H stretch), 1319, 1149 (s, S=O stretch); ^1^H NMR (400 MHz, DMSO-d_6_) δ (ppm): 7.87–7.83 (m, 4H, Ar), 7.76 (tt, *J =* 7.4 Hz, *J =* 1.2 Hz, 1H, Ar), 7.67–7.62 (m, 4H, Ar), 7.53 (s, 2H, SO_2_NH_2_), 7.29–7.24 (m, 4H, Ar), 2.35 (s, 3H, CH_3_); ^13 ^C NMR (100 MHz, DMSO-d_6_) δ (ppm):145.72, 145.09, 140.75, 140.66, 140.28, 137.83, 134.74, 130.80, 130.10, 129.44, 127.96, 127.22, 127.16, 121.18, 21.43; HRMS (ESI-MS) *m/z* 455.0856 (M + H)^+^, C_21_H_18_N_4_O_4_S_2_H^+^, calcd 455.0847.

##### 2.1.2.3. 4-(5-(4-fluorophenyl)-4-(phenylsulphonyl)-1H-1,2,3-triazol-1-yl)benzenesulphonamide (9c)

Yield 90%; off-white solid; mp: 207–209 °C; IR (ν, cm^−1^): 3317, 3225 (m, N–H stretch), 1335, 1157 (s, S=O stretch); ^1^H NMR (400 MHz, DMSO-d_6_) δ (ppm): 7.89–7.84 (m, 4H, Ar), 7.76 (t, *J =* 7.4 Hz, 1H, Ar), 7.67–7.63 (m, 4H, Ar), 7.51–7.47 (m, 4H, Ar, SO_2_NH_2_), 7.32 (t, *J =* 8.8 Hz, 2H, Ar); ^13 ^C NMR (100 MHz, DMSO-d_6_) δ (ppm): 163.63 (d, ^1^J_CF_ = 247.2 Hz), 145.82, 145.32, 140.56, 139.40, 137.69, 134.81, 133.59 (d, ^3^J_CF_ = 9 Hz), 130.13, 128.01, 127.21, 120.71 (d, ^4^J_CF_ = 4 Hz), 116.09 (d, ^2^J_CF_ = 22 Hz); HRMS (ESI-MS) *m/z* 459.0584 (M + H)^+^, C_20_H_15_FN_4_O_4_S_2_H^+^, calcd 459.0597.

##### 2.1.2.4. 4-(5-(4-chlorophenyl)-4-(phenylsulphonyl)-1H-1,2,3-triazol-1-yl)benzenesulphonamide (9d)

Yield 83%; white solid; mp: 213–215 °C; IR (ν, cm^−1^): 3348, 3271 (m, N–H stretch), 1319, 1149 (s, S=O stretch); ^1^H NMR (400 MHz, DMSO-d_6_) δ (ppm): 7.89–7.86 (m, 4H, Ar), 7.77 (tt, *J =* 7.6 Hz, *J =* 1.2 Hz, 1H, Ar), 7.68–7.63 (m, 4H, Ar), 7.57–7.44 (m, 6H, Ar, SO_2_NH_2_); ^13 ^C NMR (100 MHz, DMSO-d_6_) δ (ppm): 145.83, 145.31, 140.50, 139.19, 137.63, 136.00, 134.86, 132.91, 130.17, 129.02, 128.05, 127.24, 123.32; HRMS (ESI-MS) *m/z* 475.0332 (M + H)^+^, 477.0300 (M + H + 2)^+^, C_20_H_15_ClN_4_O_4_S_2_H^+^, calcd 475.0301.

##### 2.1.2.5. 4-(5-(4-bromophenyl)-4-(phenylsulphonyl)-1H-1,2,3-triazol-1-yl)benzenesulphonamide (9e)

Yield 88%; off-white solid; mp: 198–200 °C; IR (ν, cm^−1^): 3348, 3263 (m, N–H stretch), 1319, 1149 (s, S=O stretch); ^1^H NMR (400 MHz, DMSO-d_6_) δ (ppm): 7.90–7.87 (m, 4H, Ar), 7.78 (tt, *J =* 7.4 Hz, *J =* 1.2 Hz, 1H, Ar), 7.70–7.63 (m, 6H, Ar), 7.52 (s, 2H, SO_2_NH_2_), 7.41–7.37 (m, 2H, Ar); ^13 ^C NMR (100 MHz, DMSO-d_6_) δ (ppm): 145.86, 145.29, 140.52, 139.25, 137.64, 134.88, 133.10, 131.95, 130.19, 128.07, 127.27, 124.89, 123.72; HRMS (ESI-MS) *m/z* 518.9801 (M + H)^+^, 520.9779 (M + H + 2)^+^, C_20_H_15_BrN_4_O_4_S_2_H^+^, calcd 518.9796.

##### 2.1.2.6. 4-(5-(3-bromophenyl)-4-(phenylsulphonyl)-1H-1,2,3-triazol-1-yl)benzenesulphonamide (9f)

Yield 81%; off-white solid; mp: 282–284 °C; IR (ν, cm^−1^): 3286, 3209 (m, N–H stretch), 1335, 1149 (s, S=O stretch); ^1^H NMR (400 MHz, DMSO-d_6_) δ (ppm): 7.91–7.84 (m, 4H, Ar), 7.78 (tt, *J =* 7.4 Hz, *J =* 1.2 Hz, 1H, Ar), 7.74–7.65 (m, 6H, Ar), 7.52 (s, 2H, SO_2_NH_2_), 7.40–7.39 (m, 2H, Ar); ^13 ^C NMR (100 MHz, DMSO-d_6_) δ (ppm): 145.96, 145.45, 140.45, 138.72, 137.62, 134.91, 133.88, 133.67, 130.90, 130.16, 130.03, 128.05, 127.30, 127.25, 126.65, 121.73; HRMS (ESI-MS) *m/z* 518.9829 (M + H)^+^, 520.9806 (M + H + 2)^+^, C_20_H_15_BrN_4_O_4_S_2_H^+^, calcd 518.9796.

##### 2.1.2.7. 4-(5-(4-methoxyphenyl)-4-(phenylsulphonyl)-1H-1,2,3-triazol-1-yl)benzenesulphonamide (9g)

Yield 82%; light brown solid; mp: 235–237 °C; IR (ν, cm^−1^): 3317, 3232 (m, N–H stretch), 1319, 1149 (s, S=O stretch); ^1^H NMR (400 MHz, DMSO-d_6_) δ (ppm): 7.89–7.82 (m, 4H, Ar), 7.75 (tt, *J =* 7.4 Hz, *J =* 1.2 Hz, 1H, Ar), 7.66–7.62 (m, 4H, Ar), 7.53 (s, 2H, SO_2_NH_2_), 7.34–7.30 (m, 2H, Ar), 7.02–6.98 (m, 2H, Ar), 3.80 (s, 3H, OCH_3_); ^13 ^C NMR (100 MHz, DMSO-d_6_) δ (ppm): 161.16, 145.68, 145.02, 140.71, 140.20, 137.93, 134.74, 132.52, 130.10, 127.96, 127.20, 127.18, 115.81, 114.40, 55.77; HRMS (ESI-MS) *m/z* 471.0828 (M + H)^+^, C_21_H_18_N_4_O_5_S_2_H^+^, calcd 471.0797.

##### 2.1.2.8. 4-(5-(3-bromo-4-methoxyphenyl)-4-(phenylsulphonyl)-1H-1,2,3-triazol-1-yl)benzenesulphonamide (9h)

Yield 82%; off-white solid; mp: 210–212 °C; IR (ν, cm^−1^): 3294, 3217 (m, N–H stretch), 1335, 1149 (s, S=O stretch); ^1^H NMR (400 MHz, DMSO-d_6_) δ (ppm): 7.91–7.88 (m, 2H, Ar), 7.85–7.83 (m, 2H, Ar), 7.77 (t, *J =* 7.4 Hz, 1H, Ar), 7.68–7.64 (m, 5H, Ar), 7.53 (s, 2H, SO_2_NH_2_), 7.36 (dd, *J =* 8.4 Hz, *J =* 2.4 Hz, 1H, Ar), 7.16 (d, *J =* 8.4 Hz, 1H, Ar), 3.89 (s, 3H, OCH_3_); ^13 ^C NMR (100 MHz, DMSO-d_6_) δ (ppm): 157.30, 145.81, 145.27, 140.58, 138.85, 137.78, 135.34, 134.83, 131.98, 130.12, 128.00, 127.28, 127.24, 117.34, 112.77, 110.69, 56.95; HRMS (ESI-MS) *m/z* 548.9904 (M + H)^+^, 550.9882 (M + H + 2)^+^, C_21_H_17_BrN_4_O_5_S_2_H^+^, calcd 548.9902.

##### 2.1.2.9. 4-(5-(4-nitrophenyl)-4-(phenylsulphonyl)-1H-1,2,3-triazol-1-yl)benzenesulphonamide (9i)

Yield 94%; pale yellow solid; mp: 278–280 °C; IR (ν, cm^−1^): 3356, 3240 (m, N–H stretch), 1342, 1157 (s, S=O stretch); ^1^H NMR (400 MHz, DMSO-d_6_) δ (ppm): 8.34–8.30 (m, 2H, Ar), 7.91–7.87 (m, 4H, Ar), 7.81–7.77 (m, 3H, Ar), 7.70–7.65 (m, 4H, Ar), 7.50 (s, 2H, SO_2_NH_2_); ^13 ^C NMR (100 MHz, DMSO-d_6_) δ (ppm): 149.11, 145.96, 145.65, 140.34, 138.43, 137.46, 134.99, 132.82, 131.19, 130.25, 128.16, 127.33, 127.26, 123.83; HRMS (ESI-MS) *m/z* 486.0549 (M + H)^+^, C_20_H_15_N_5_O_6_S_2_H^+^, calcd 486.0542.

##### 2.1.2.10. 4-(5-(3-nitrophenyl)-4-(phenylsulphonyl)-1H-1,2,3-triazol-1-yl)benzenesulphonamide (9j)

Yield 83%; off-white solid; mp: 186–188 °C; IR (ν, cm^−1^): 3371, 3310 (m, N–H stretch), 1342, 1157 (s, S=O stretch); ^1^H NMR (400 MHz, DMSO-d_6_) δ (ppm): 8.44 (t, *J =* 2 Hz, 1H, Ar), 8.37 (ddd, *J =* 8.4 Hz, *J =* 2.4 Hz, *J =* 1.2 Hz, 1H, Ar), 7.89–7.83 (m, 5H, Ar), 7.80–7.72 (m, 2H, Ar), 7.70–7.65 (m, 4H, Ar), 7.51 (s, 2H, SO_2_NH_2_); ^13 ^C NMR (100 MHz, DMSO-d_6_) δ (ppm): 147.75, 145.97, 145.65, 140.35, 138.19, 137.45, 137.38, 134.96, 130.57, 130.21, 128.11, 127.33, 127.29, 126.42, 126.27, 125.84; HRMS (ESI-MS) *m/z* 486.0547 (M + H)^+^, C_20_H_15_N_5_O_6_S_2_H^+^, calcd 486.0542.

##### 2.1.2.11. 4-(5-([1,1′-biphenyl]-4-yl)-4-(phenylsulphonyl)-1H-1,2,3-triazol-1-yl)benzenesulphonamide (9k)

Yield 83%; off-white solid; mp: 214–216 °C; IR (ν, cm^−1^): 3371, 3286 (m, N–H stretch), 1335, 1149 (s, S=O stretch); ^1^H NMR (400 MHz, DMSO-d_6_) δ (ppm): 7.89–7.87 (m, 4H, Ar), 7.79–7.74 (m, 5H, Ar), 7.70–7.63 (m, 4H, Ar), 7.52–7.48 (m, 6H, Ar, SO_2_NH_2_), 7.44–7.40 (m, 1H, Ar); ^13 ^C NMR (100 MHz, DMSO-d_6_) δ (ppm): 145.80, 145.32, 142.24, 140.59, 139.96, 139.94, 139.11, 137.85, 134.82, 131.62, 130.13, 129.55, 128.70, 128.07, 127.27, 127.26, 126.88, 123.24; HRMS (ESI-MS) *m/z* 517.1039 (M + H)^+^, C_26_H_20_N_4_O_4_S_2_H^+^, calcd 517.1004.

##### 2.1.2.12. 3-(5-phenyl-4-(phenylsulphonyl)-1H-1,2,3-triazol-1-yl)benzenesulphonamide (10a)

Yield 97%; white solid; mp: 172–174 °C; IR (ν, cm^−1^): 3371, 3286 (m, N–H stretch), 1311, 1142 (s, S=O stretch); ^1^H NMR (400 MHz, DMSO-d_6_) δ (ppm): 8.01 (t, *J =* 2 Hz, 1H, Ar), 7.91 (dt, *J =* 7.6 Hz, *J =* 1.2 Hz, 1H, Ar), 7.85–7.83 (m, 2H, Ar), 7.75 (tt, *J =* 7.6 Hz, *J =* 1.2 Hz, 1H, Ar), 7.66–7.60 (m, 3H, Ar), 7.55–7.48 (m, 4H, Ar, SO_2_NH_2_), 7.45–7.39 (m, 4H, Ar); ^13 ^C NMR (100 MHz, DMSO-d_6_) δ (ppm): 145.79, 145.10, 140.68, 140.32, 135.68, 134.73, 130.94, 130.61, 130.08, 129.57, 128.79, 127.96, 127.68, 124.24 123.95; HRMS (ESI-MS) *m/z* 441.0692 (M + H)^+^, C_20_H_16_N_4_O_4_S_2_H^+^, calcd 441.0691.

##### 2.1.2.13. 3-(4-(phenylsulphonyl)-5-(p-tolyl)-1H-1,2,3-triazol-1-yl)benzenesulphonamide (10b)

Yield 90%; off-white solid; mp: 186–188 °C; IR (ν, cm^−1^): 3310, 3240 (m, N–H stretch), 1311, 1165 (s, S=O stretch); ^1^H NMR (400 MHz, DMSO-d_6_) δ (ppm): 8.02 (t, *J =* 2 Hz, 1H, Ar), 7.92 (dt, *J =* 7.6 Hz, *J =* 1.2 Hz, 1H, Ar), 7.85–7.83 (m, 2H, Ar), 7.75 (tt, *J =* 7.4 Hz, *J =* 1.2 Hz, 1H, Ar), 7.66–7.60 (m, 3H, Ar), 7.54–7.52 (m, 3H, Ar, SO_2_NH_2_), 7.30–7.23 (m, 4H, Ar), 2.34 (s, 3H, CH_3_); ^13 ^C NMR (100 MHz, DMSO-d_6_) δ (ppm): 145.79, 144.99, 140.76, 140.71, 140.42, 135.75, 134.71, 130.82, 130.64, 130.08, 129.65, 129.41, 127.95, 127.68, 124.03, 121.16, 21.46; HRMS (ESI-MS) *m/z* 455.0847 (M + H)^+^, C_21_H_18_N_4_O_4_S_2_H^+^, calcd 455.0847.

##### 2.1.2.14. 3-(5-(4-fluorophenyl)-4-(phenylsulphonyl)-1H-1,2,3-triazol-1-yl)benzenesulphonamide (10c)

Yield 82%; light brown solid; mp: 128–130 °C; IR (ν, cm^−1^): 3379, 3279 (m, N–H stretch), 1319, 1149 (s, S=O stretch); ^1^H NMR (400 MHz, DMSO-d_6_) δ (ppm): 8.00 (t, *J =* 1.6 Hz, 1H, Ar), 7.93–7.91 (m, 1H, Ar), 7.86–7.84 (m, 2H, Ar), 7.76 (tt, *J =* 7.4 Hz, *J =* 1.2 Hz, 1H, Ar), 7.67–7.62 (m, 3H, Ar), 7.57–7.48 (m, 5H, Ar, SO_2_NH_2_), 7.32–7.28 (m, 2H, Ar); ^13 ^C NMR (100 MHz, DMSO-d_6_) δ (ppm): 163.60 (d, ^1^J_CF_ = 246 Hz), 145.76, 145.15, 140.60, 139.53, 135.58, 134.76, 133.57 (d, ^3^J_CF_ = 9 Hz), 130.66, 130.10, 129.59, 127.98, 127.71, 123.96, 120.66 (d, ^4^J_CF_ = 3 Hz), 116.03 (d, ^2^J_CF_ = 23 Hz); HRMS (ESI-MS) *m/z* 459.0600 (M + H)^+^, C_20_H_15_FN_4_O_4_S_2_H^+^, calcd 459.0597.

##### 2.1.2.15. 3-(5-(4-chlorophenyl)-4-(phenylsulphonyl)-1H-1,2,3-triazol-1-yl)benzenesulphonamide (10d)

Yield 91%; off-white solid; mp: 123–125 °C; IR (ν, cm^−1^): 3302, 3232 (m, N–H stretch), 1319, 1165 (s, S=O stretch); ^1^H NMR (400 MHz, DMSO-d_6_) δ (ppm): 8.03 (t, *J =* 2 Hz, 1H, Ar), 7.93 (dt, *J =* 8 Hz, *J =* 1.2 Hz, 1H, Ar), 7.88–7.86 (m, 2H, Ar), 7.77 (tt, *J =* 7.4 Hz, *J =* 1.2 Hz, 1H, Ar), 7.68–7.63 (m, 3H, Ar), 7.58–7.52 (m, 5H, Ar, SO_2_NH_2_), 7.48–7.45 (m, 2H, Ar); ^13 ^C NMR (100 MHz, DMSO-d_6_) δ (ppm): 145.82, 145.21, 140.59, 139.36, 135.99, 135.54, 134.81, 132.90, 130.71, 130.13, 129.63, 128.97, 128.02, 127.80, 124.05, 123.30; HRMS (ESI-MS) *m/z* 475.0301 (M + H)^+^, 477.0270 (M + H + 2)^+^, C_20_H_15_ClN_4_O_4_S_2_H^+^, calcd 475.0301.

##### 2.1.2.16. 3-(5-(4-bromophenyl)-4-(phenylsulphonyl)-1H-1,2,3-triazol-1-yl)benzenesulphonamide (10e)

Yield 69%; off-white solid; mp: 96–98 °C; IR (ν, cm^−1^): 3356, 3286 (m, N–H stretch), 1335, 1157 (s, S=O stretch); ^1^H NMR (400 MHz, DMSO-d_6_) δ (ppm): 8.04 (t, *J =* 1.8 Hz, 1H, Ar), 7.93 (dt, *J =* 8 Hz, *J =* 1.4 Hz, 1H, Ar), 7.88–7.86 (m, 2H, Ar), 7.77 (tt, *J =* 7.6 Hz, *J =* 1.2 Hz, 1H, Ar), 7.68–7.63 (m, 5H, Ar), 7.57–7.53 (m, 3H, Ar, SO_2_NH_2_), 7.41–7.39 (m, 2H, Ar); ^13 ^C NMR (100 MHz, DMSO-d_6_) δ (ppm): 145.81, 145.13, 140.57, 139.41, 135.53, 134.81, 133.07, 131.87, 130.70, 130.13, 129.64, 128.02, 127.80, 124.84, 124.07, 123.68; HRMS (ESI-MS) *m/z* 518.9827 (M + H)^+^, 520.9807 (M + H + 2)^+^, C_20_H_15_BrN_4_O_4_S_2_H^+^, calcd 518.9796.

##### 2.1.2.17. 3-(5-(3-bromophenyl)-4-(phenylsulphonyl)-1H-1,2,3-triazol-1-yl)benzenesulphonamide (10f)

Yield 83%; off-white solid; mp: 97–99 °C; IR (ν, cm^−1^): 3317, 3240 (m, N–H stretch), 1335, 1157 (s, S=O stretch); ^1^H NMR (400 MHz, DMSO-d_6_) δ (ppm): 8.04 (t, *J =* 2 Hz, 1H, Ar), 7.93 (dt, *J =* 7.6 Hz, *J =* 1.2 Hz, 1H, Ar), 7.86–7.83 (m, 2H, Ar), 7.77 (tt, *J =* 7.4 Hz, *J =* 1.2 Hz, 1H, Ar), 7.71–7.64 (m, 5H, Ar), 7.60–7.53 (m, 3H, Ar, SO_2_NH_2_), 7.43–7.36 (m, 2H, Ar); ^13 ^C NMR (100 MHz, DMSO-d_6_) δ (ppm): 145.85, 145.28, 140.50, 138.83, 135.50, 134.84, 133.83, 133.64, 130.82, 130.63, 130.10, 130.04, 129.59, 128.00, 127.84, 126.61, 124.04, 121.67; HRMS (ESI-MS) *m/z* 518.9771 (M + H)^+^, 520.9750 (M + H + 2)^+^, C_20_H_15_BrN_4_O_4_S_2_H^+^, calcd 518.9796.

##### 2.1.2.18. 3-(5-(4-methoxyphenyl)-4-(phenylsulphonyl)-1H-1,2,3-triazol-1-yl)benzenesulphonamide (10g)

Yield 72%; off-white solid; mp: 161–163 °C; IR (ν, cm^−1^): 3379, 3279 (m, N–H stretch), 1358, 1165 (s, S=O stretch); ^1^H NMR (400 MHz, DMSO-d_6_) δ (ppm): 8.00 (t, *J =* 2 Hz, 1H, Ar), 7.92–7.90 (m, 1H, Ar), 7.84–7.82 (m, 2H, Ar), 7.75 (tt, *J =* 7.4 Hz, *J =* 1.2 Hz, 1H, Ar), 7.66–7.61 (m, 3H, Ar), 7.54–7.51 (m, 3H, Ar, SO_2_NH_2_), 7.33–7.29 (m, 2H, Ar), 6.99–6.96 (m, 2H, Ar), 3.79 (s, 3H, OCH_3_); ^13 ^C NMR (100 MHz, DMSO-d_6_) δ (ppm): 161.13, 145.78, 144.88, 140.77, 140.32, 135.81, 134.69, 132.51, 130.64, 130.07, 129.59, 127.92, 127.60, 123.94, 115.75, 114.36, 55.75; HRMS (ESI-MS) *m/z* 471.0796 (M + H)^+^, C_21_H_18_N_4_O_5_S_2_H^+^, calcd 471.0797.

##### 2.1.2.19. 3-(5-(3-bromo-4-methoxyphenyl)-4-(phenylsulphonyl)-1H-1,2,3-triazol-1-yl)benzenesulphonamide (10h)

Yield 69%; white solid; mp: 231–233 °C; IR (ν, cm^−1^): 3394, 3256 (m, N–H stretch), 1327, 1149 (s, S=O stretch); ^1^H NMR (400 MHz, DMSO-d_6_) δ (ppm): 8.05 (s, 1H, Ar), 7.93 (d, *J =* 7.6 Hz, 1H, Ar), 7.85–7.75 (m, 3H, Ar), 7.67–7.56 (m, 7H, Ar, SO_2_NH_2_), 7.37 (d, *J =* 8.8 Hz, 1H, Ar), 7.15 (d, *J =* 8.8 Hz, 1H, Ar), 3.88 (s, 3H, OCH_3_); ^13 ^C NMR (100 MHz, DMSO-d_6_) δ (ppm): 157.26, 145.79, 145.09, 140.63, 138.96, 135.66, 135.32, 134.76, 131.99, 130.67, 130.07, 129.64, 127.95, 127.74, 124.06, 117.30, 112.71, 110.63, 56.91; HRMS (ESI-MS) *m/z* 548.9902 (M + H)^+^, 550.9880 (M + H + 2)^+^, C_21_H_17_BrN_4_O_5_S_2_H^+^, calcd 548.9902.

##### 2.1.2.20. 3-(5-(4-nitrophenyl)-4-(phenylsulphonyl)-1H-1,2,3-triazol-1-yl)benzenesulphonamide (10i)

Yield 81%; pale yellow solid; mp: 182–184 °C; IR (ν, cm^−1^): 3356, 3286 (m, N–H stretch), 1342, 1157 (s, S=O stretch); ^1^H NMR (400 MHz, DMSO-d_6_) δ (ppm): 8.32–8.29 (m, 2H, Ar), 8.04–8.03 (m, 1H, Ar), 7.94–7.89 (m, 3H, Ar), 7.80–7.76 (m, 3H, Ar), 7.70–7.60 (m, 4H, Ar), 7.53 (s, 2H, SO_2_NH_2_); ^13 ^C NMR (100 MHz, DMSO-d_6_) δ (ppm): 149.08, 145.84, 145.52, 140.44, 138.63, 135.38, 134.93, 132.83, 131.23, 130.84, 130.22, 129.69, 128.14, 127.96, 124.08, 123.77; HRMS (ESI-MS) *m/z* 486.0549 (M + H)^+^, C_20_H_15_N_5_O_6_S_2_H^+^, calcd 486.0542.

##### 2.1.2.21. 3-(5-(3-nitrophenyl)-4-(phenylsulphonyl)-1H-1,2,3-triazol-1-yl)benzenesulphonamide (10j)

Yield 98%; white solid; mp: 239–241 °C; IR (ν, cm^−1^): 3310, 3232 (m, N–H stretch), 1327, 1149 (s, S=O stretch); ^1^H NMR (400 MHz, DMSO-d_6_) δ (ppm): 8.43 (t, *J =* 2 Hz, 1H, Ar), 8.35 (ddd, *J =* 8.4 Hz, *J =* 2.4 Hz, *J =* 1.2 Hz, 1H, Ar), 8.04–8.02 (m, 1H, Ar), 7.93–7.86 (m, 4H, Ar), 7.79–7.71 (m, 2H, Ar), 7.68–7.63 (m, 4H, Ar), 7.53 (s, 2H, SO_2_NH_2_); ^13 ^C NMR (100 MHz, DMSO-d_6_) δ (ppm): 147.69, 145.82, 145.49, 140.43, 138.33, 137.47, 135.37, 134.89, 130.76, 130.51, 130.16, 129.69, 128.07, 127.87, 126.37, 126.27, 125.79, 124.07; HRMS (ESI-MS) *m/z* 486.0541 (M + H)^+^, C_20_H_15_N_5_O_6_S_2_H^+^, calcd 486.0542.

##### 2.1.2.22. 3-(5-([1,1′-biphenyl]-4-yl)-4-(phenylsulphonyl)-1H-1,2,3-triazol-1-yl)benzenesulphonamide (10k)

Yield 71%; off-white solid; mp: 74–76 °C; IR (ν, cm^−1^): 3379, 3325 (m, N–H stretch), 1335, 1157 (s, S=O stretch); ^1^H NMR (400 MHz, DMSO-d_6_) δ (ppm): 8.07–8.06 (t, *J =* 2 Hz, 1H, Ar) 7.94–7.87 (m, 3H, Ar), 7.78–7.73 (m, 5H, Ar), 7.67–7.58 (m, 4H, Ar), 7.55 (s, 2H, SO_2_NH_2_), 7.52–7.48 (m, 4H, Ar), 7.42 (tt, *J =* 7.4 Hz, *J =* 1.2 Hz, 1H, Ar); ^13 ^C NMR (100 MHz, DMSO-d_6_) δ (ppm): 145.81, 145.16, 142.24, 140.64, 140.07, 139.20, 135.73, 134.76, 131.60, 130.69, 130.09, 129.63, 129.51, 128.65, 128.03, 127.72, 127.29, 126.86, 124.02, 123.19; HRMS (ESI-MS) *m/z* 517.0992 (M + H)^+^, C_26_H_20_N_4_O_4_S_2_H^+^, calcd 517.1004.

### 2.2. *In vitro* CA inhibition assay

An Sx.18Mv-R Applied Photophysics (Oxford, UK) stopped-flow instrument was used to assay the catalytic activity of various CA isozymes for CO_2_ hydration reaction.^21^

### 2.3. Biological assays

#### 2.3.1. *In vitro* culture and treatment of testicular cells/tissue

Goat (*Capra hircus*) testicular tissues were procured from slaughterhouses of Chandigarh (30°43′ N, 76°12′ E) and around Kurukshetra (29°6′ N, 76°50′ E) in 0.9% normal saline at 4 °C. The testicular cells/tissues were cultured *in vitro* in media-TCM199 and exposed to 10 µM concentration of given compounds for 6 h in a CO_2_ incubator with 5% CO_2_ and 95% humidity at 38 °C temperature.^22^^,^^23^

#### 2.3.2. Methyl thiazolyl tetrazolium (MTT) assay

Testicular cells were cultured in 96-well plates. Wells for blank control were without any cells and contained MTT [3-(4,5-dimethylthiazol-2-yl)-2,5-diphenyl-tetrazolium-bromide)], culture medium, and DMSO. The remaining wells were seeded with testicular cells, which were treated with given compounds at 10 µM concentration for 6 h. Post treatment, 10 µL of MTT stock solution was added to each well, and the reaction mixture was incubated for 2 h. After that 200 µL of DMSO was added in each well, followed by shaking for 10 min. Absorbance of reaction mixture was taken at 470 nm.

#### 2.3.3. Classical histology

Testicular tissues from both treated and untreated control groups were fixed in Bouin’s fixative for 48 h, washed, dehydrated through graded series of ethanol, and embedded in paraffin wax at 60 °C. After trimming the paraffin-embedded tissue blocks, sectioning of tissue was done at 5 µm using rotary microtome. Eventually, the sections were stretched and stained with haematoxylin and eosin (H & E) stain,^24^ followed by their observation under light microscope (Olympus, Tokyo, Japan).

#### 2.3.4. Fluorescence assay (ethidium bromide/acridine orange staining) for apoptosis detection

Ethidium bromide and acridine orange (EB/AO) staining was used to determine the cyto-genotoxic nature of given compounds in testicular cells.^25^ Briefly, the testicular cell suspension prepared from the treated testicular tissue was mixed with equal volume of EB/AO differential stain on a glass slide and covered with a coverslip. Observation and quantification of apoptotic cells were done with fluorescence microscope (Olympus, Japan) using filters of 500–525 nm. Cells appearing bright green were marked as normal healthy cells, while cells displaying orange-red fluorescence were marked as apoptotic.

#### 2.3.5. Analysis of oxidative stress

Tissues from the treated and control groups were homogenised in 1 ml phospate buffer saline (pH 7.2) to make the testicular homogenate for oxidative stress measurements induced by the given compounds in testicular tissue.

##### 2.3.5.1. Thiobarbituric acid-reactive substances (TBARS) assay

TBARS (e.g. malondialdehyde) are used as oxidative stress marker and the level of malondialdehyde (MDA) in testicular homogenate was measured according to earlier reported method.^26^ Briefly, the tissue homogenate was added to 2 ml of thiobarbituric acid (TBA)-HCl reagent, 1 ml of trichloroacetic acid and this mixture was then heated on water bath for 15 min. The MDA levels (µmoles/g wet tissue weight) were measured spectrophotometrically at 535 nm using extinction coefficient of MDA, i.e. 1.56 × 10^5^ mol^−1 ^cm^−1^.

##### 2.3.5.2. Ferric reducing antioxidant power (FRAP) assay

Briefly, the testicular homogenate (100 µL) was mixed with 3 ml of freshly prepared FRAP reagent and incubated for 4 min at 37 °C in water bath. Absorbance of reaction mixture was measured at 593 nm wavelength using IMPLEN nanospectrophotometer (Bavaria, Germany). The change in absorption is directly proportional to the combined or total reduction of the electron-donating antioxidants present in the reaction mixture.^27^

#### 2.3.6. Statistical analysis

All the experiments were carried out in triplicates to ensure biological reproducibility, and data are expressed as mean ± standard error of mean (SEM) using statistical software SPSS 16 (SPSS, Chicago, IL) to ensure statistical validity.

## 3. Results and discussion

### 3.1. Chemistry

**Scheme 1.**
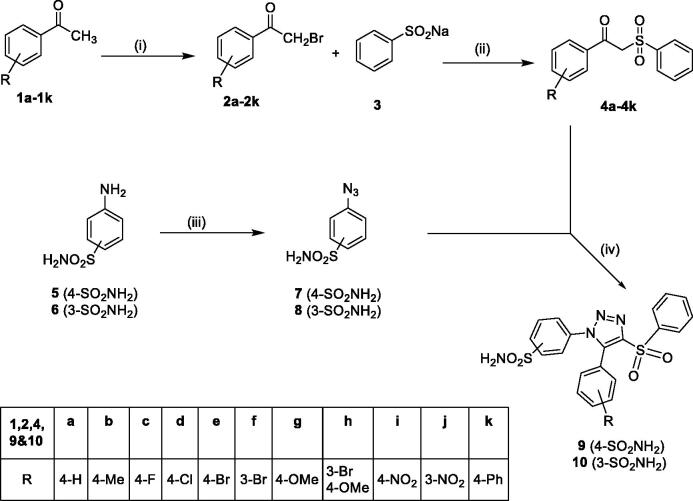
Reagents and conditions: (i) Br_2_, acetic acid; (ii) 1,2-dimethoxyethane, tetrabutylammonium iodide, reflux; (iii) (a) HCl, NaNO_2_, 0 *°C* and (b) NaN_3_, 0 *°C*; and (iv) DMSO, piperidine.

The synthesis of two subsets, 4-(5-aryl-(4-phenylsulphonyl)-1*H*-1,2,3-triazol-1-yl)benzenesulphonamide derivatives **9a–9k** and 3-(5-aryl-(4-phenylsulpnonyl)-1*H*-1,2,3-triazol-1-yl)benzenesulphonamide derivatives **10a–10k**, consisting of total 22 novel target compounds was achieved by adopting the synthetic route outlined in Scheme 1. Differently substituted phenacyl bromides **2a–2k** were prepared by the bromination of corresponding acetophenone derivatives **1a–1k** using bromine in acetic acid. The synthesised phenacyl bromides **2a–2k** were allowed to react with sodium benzene sulphinate **3**^28^ under refluxing conditions in 1,2-dimethoxyethane to give the corresponding key intermediates β-ketosulphones **4a–4k.** Compounds **7** and **8** were synthesised by the diazotisation of corresponding 4- and 3-aminobenzenesulphonamide (**5–6)** in cold conditions followed by the addition of sodium azide. Further, deploying the organocatalysed [3 + 2] cycloaddition approach for the synthesis of 1,2,3-triazoles,^29^ β-ketosulphone derivatives **4a–4k** were reacted with 4- and 3-azidobenzenesulphonamides **7** and **8** in DMSO using piperidine as organo-catalyst to afford respective target compounds **9a–9k** and **10a–10k**.

The target compounds thus obtained were characterised with the help of spectroscopic techniques including FT-IR, ^1^H-NMR, ^13^C-NMR and finally by HRMS analysis. The spectral analysis was in full agreement with the postulated structures. In IR spectra, N–H stretch of SO_2_NH_2_ group was observed in the absorption range of 3200–3400 cm^−1^. Strong bands of S=O stretch for SO_2_ and SO_2_NH_2_ groups were noted in the absorption range of 1358–1311 cm^−1^ and 1165–1142 cm^−1^. In ^1^H-NMR, the aromatic protons were observed at the expected values in accordance with the proposed structures. In most of the compounds, the signal for two protons of SO_2_NH_2_ group was observed to be merged with the aromatic envelop and in some of the compounds a singlet was noted in the narrow range at δ 7.50 to 7.55.

### 3.2. CA inhibition studies

All the 22 newly synthesised compounds **9**–**10** were evaluated for CA inhibition potential against cytosolic isoforms hCA I and II as well as cell membrane associated isoforms hCA IV and IX by using stopped-flow CO_2_ hydrase assay method.^21^ Clinically used drug acetazolamide (AAZ) was used as the reference drug for comparing the extent of inhibition of target compounds. From the inhibition profile of target compounds **9–10** displayed in Table 1, the following insights can be drawn regarding the CA inhibitory properties:

**Table 1.** Inhibition profile of newly synthesised compounds **9–10** against hCA I, II, IV, and IX isoforms compared with the standard inhibitor acetazolamide (AAZ) and selectivity index for isoforms I/IX and II/IX.

Compounds	R	K_I_(nM)^a^				Selectivity index	
		hCA I	hCA II	hCA IV	hCA IX	I/IX	II/IX
**9a**	4-H	97.9	9.6	148.8	21.5	4.55	0.45
**9b**	4-CH_3_	766.8	7.8	82.1	30.9	25.06	0.25
**9c**	4-F	264.1	6.6	86.1	66.0	4.00	0.10
**9d**	4-Cl	4629	56.7	526.5	25.1	184.42	2.26
**9e**	4-Br	7838	220.5	800.7	30.1	260.40	7.32
**9f**	3-Br	433.5	9.2	94.8	27.1	16.00	0.34
**9g**	4-OCH_3_	7837	6.2	492.7	24.9	314.73	0.25
**9h**	3-Br, 4-OCH_3_	9349	37.6	1215	28.8	324.62	1.30
**9i**	4-NO_2_	284.8	4.9	84.7	32.4	8.79	0.15
**9j**	3-NO_2_	45.4	6.1	89.6	26.3	1.73	0.23
**9k**	4-Ph	3543	833.3	414.5	24.8	142.86	33.60
**10a**	4-H	607.0	161.2	78.6	26.0	23.35	6.20
**10b**	4-CH_3_	566.3	21.8	347.1	21.2	26.71	1.03
**10c**	4-F	454.1	119.3	78.9	22.8	19.92	5.23
**10d**	4-Cl	695.7	58.4	91.8	16.4	42.42	3.56
**10e**	4-Br	635.8	138.1	80.1	25.1	25.33	5.50
**10f**	3-Br	689.2	369.9	84.3	28.6	24.10	12.93
**10g**	4-OCH_3_	508.5	44.3	84.0	24.1	21.10	1.84
**10h**	3-Br, 4-OCH_3_	8033	648.7	6048	22.8	352.32	28.45
**10i**	4-NO_2_	868.4	35.2	64.4	27.0	32.16	1.30
**10j**	3-NO_2_	6895	884.4	1684	26.7	258.24	33.12
**10k**	4-Ph	2057	210.8	848.6	22.6	91.02	9.33
**AAZ**		250.0	12.1	74.0	25.8	9.69	0.47

^a^Mean from three different assays, by a stopped-flow technique (errors were in the range of ± 5–10% of the reported values).

1. In case of hCA I isoform, the target compounds exhibited the range of K_I_ (inhibition constant) values from 45.4 nM to 9.34 µM. In addition to this, two compounds **9a** and **9j** showed better inhibition as compared to AAZ (K_I_ = 250 nM) displaying K_I_ value < 100 nM. Compounds **9h** and **10h** possessing 3,4-disubstituted phenyl ring appended at 5-position of 1,2,3-triazole scaffold, amongst all other derivatives having only monosubstituted phenyl rings, were found to be the least potent inhibitors of hCA I in their respective subsets with K_I_ values 9.34 and 8.03 µM respectively.
2. The range of K_I_ values for hCA II was from 4.9 to 884.4 nM with seven compounds of subset **9a–9k** being more potent inhibitors than reference drug AAZ (K_I_ = 12.1 nM). All the three-substituted benzenesulphonamide derivatives exhibited weaker inhibition against hCA II in comparison to four-substituted analogues except two compounds **10e** and **10k.**
3. For hCA IV, the range of K_I_ values was found to be from 64.4 nM to 6.04 µM. Compound **10i** was the most potent and only compound showing better inhibition as compared to reference drug AAZ (K_I_ = 74.0 nM). Following the same outcome as that for hCA I inhibition, compounds **9h** and **10h** were found to be the least effective inhibitors of hCA IV among their respective subsets.
4. Interestingly, all the target compounds were low nanomolar inhibitors of tumour-associated isoform hCA IX with K_i_ values ranging from 16.4 to 66.0 nM. Eleven compounds **9a, 9d, 9g, 9k, 10b–e, 10g, 10h,** and **10k** out of total 22 newly synthesised compounds exhibited better inhibition potential against hCA IX in comparison to reference drug AAZ (K_I_ = 25.8 nM). The compound **10d** containing 4-chlorophenyl ring at 5-position of 1,2,3-triazole ring was found to be the most potent compound exhibiting K_I_ = 16.4 nM.
5. On examining the selectivity index, seven compounds **9d**, **9e**, **9g**, **9h**, **9k**, **10h,** and **10j** were found to be more than 100-fold selective for hCA IX over ubiquitous isoform hCA I. Also compounds **9h** and **10h** showed highest selectivity among all the derivatives of respective subsets and the results indicated that di-substitution of the phenyl ring appended at 5-position of 1,2,3-triazole scaffold accounts for this desired selectivity among tested hCA isoforms.
6. All the three-substituted benzenesulphonamide derivatives, except two compounds **10e** and **10k,** exhibited higher selectivity for hCA IX over hCA II in comparison to their four-substituted analogues. Moreover, **9k** and **10j** were found to be more than 30-fold selective for tumour-associated isoform hCA IX over hCA II, also exhibiting highest selectivity among their respective subsets.
7. In general, it is noteworthy that most of the newly synthesised target compounds **9–10** displayed low nanomolar as well as more potent inhibition as compared to the standard drug AAZ. Additionally, these compounds exhibited desirable isoform-selective inhibition particularly for tumour-associated isoform hCA IX over other tested isoforms hCA I, II and IV.

### 3.3. Biological assessment for apoptosis induction

Based on the inhibitory activity against tumour-associated isoform hCA IX and selectivity over off-target isoforms hCA I and II, four compounds **9k, 10d, 10h,** and **10j** were selected for assessing their apoptotic induction potential by performing *in vitro* studies in testicular cells of goat (*Capra hircus*). These compounds were tested for cytotoxicity/cell viability analysis, histopathological studies, oxidative stress evaluation, fluorescence assay and apoptosis quantification in order to examine their potential to induce apoptosis.

#### 3.3.1. Cell viability analysis

**Figure 2.**
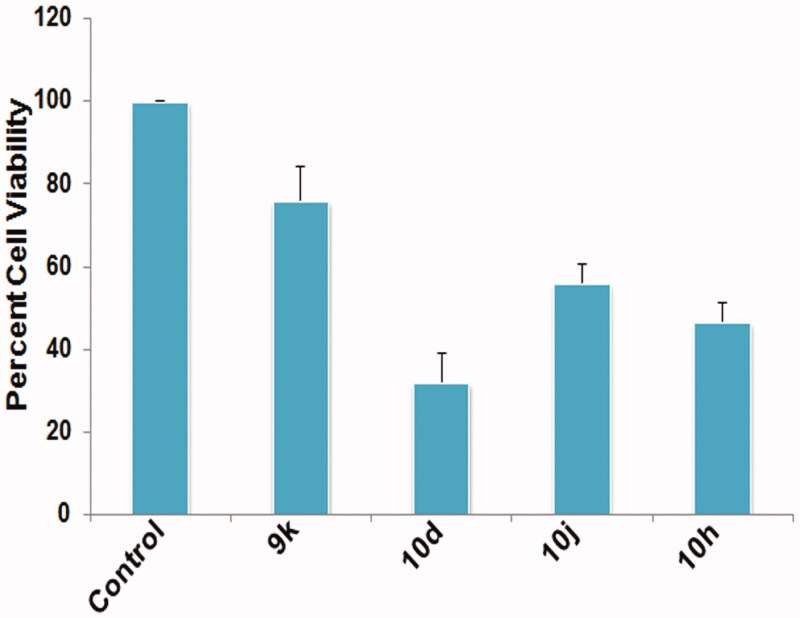
Graph depicting percent cell viability of testicular cells post treatment with different compounds at 10 μM concentration for 6 h duration. Data are expressed as mean ± SEM using statistical software SPSS 16.

The cytotoxic nature of given compounds was assessed by performing MTT assay. Results revealed a significant (*p* < .05) reduction in viability of testicular cells after treatment with the given compounds (Figure 2). However, the maximum relative decrease in viability of cells was noticed in group treated with compounds **10d** and **10h** with percent cell viability 32 ± 6.92 and 46.66 ± 4.80, respectively, in comparison to the control group.

#### 3.3.2. Histopathological observation

**Figure 3.**
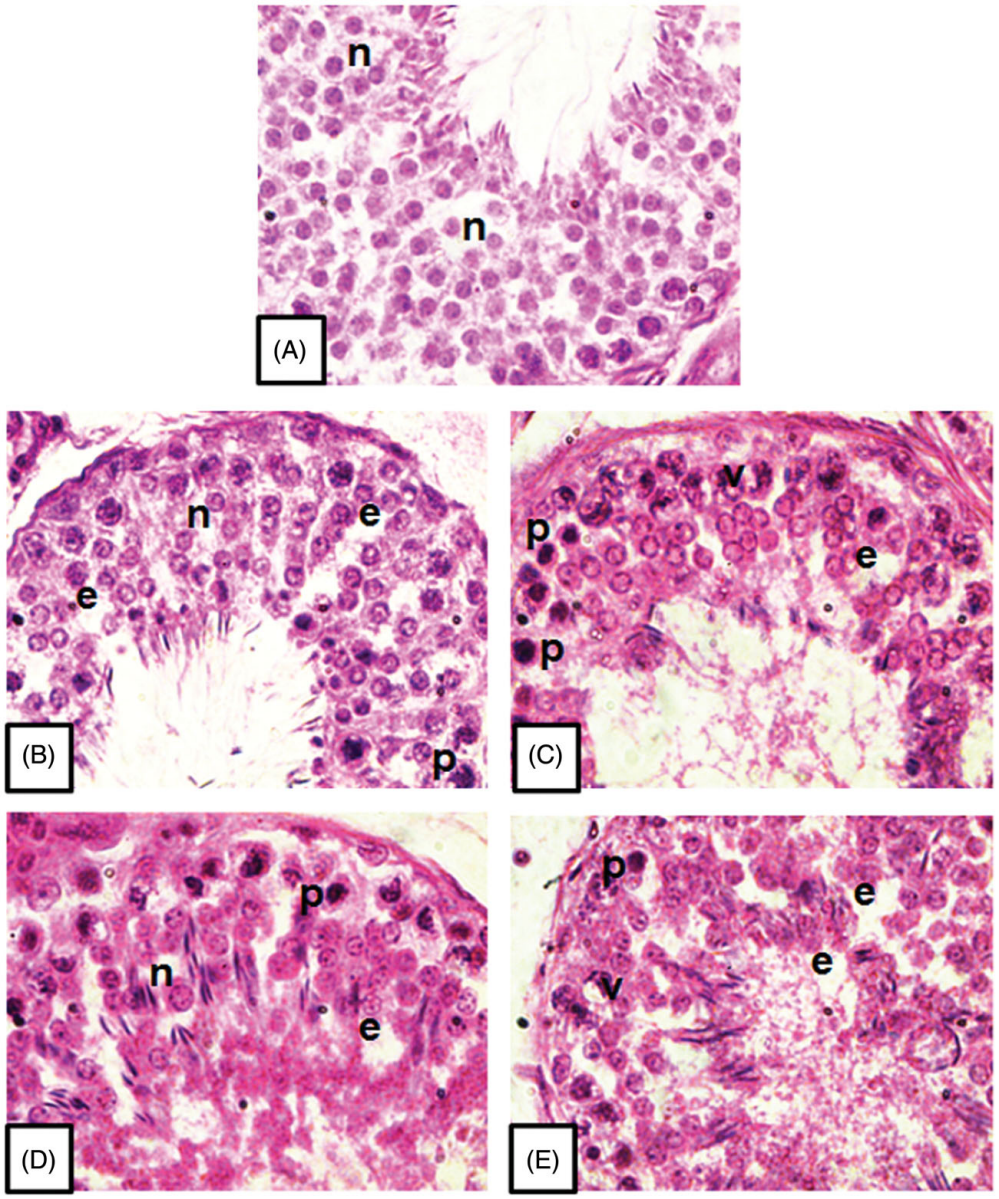
Microphotographs of testicular tissue showing histopathological alterations in testicular cells post treatment with different compounds (B–E) at 10 μM concentration for 6 h duration in comparison to the control (A). n: normal healthy cells; p: pyknotic nuclei; e: empty spaces, v: cytoplasmic vacuolisation (H & E stain, 1000×).

Outcomes of histopathological study showed different kinds of structural abnormalities in normal architecture of testicular tissue, which are characteristics of apoptosis. Treatment with the given compounds induced condensation of chromatin material in testicular cells, which appeared as darkly stained pyknotic nuclei, empty spaces between testicular germ cells, and cytoplasmic vacuolisation of testicular cells (Figure 3(B–E)). On the other hand, in the control group, the testicular tissue appeared normal in architecture with less apoptotic nuclei and homogeneous arrangement of all the spermatogenic cells within the seminiferous tubule (Figure 3(A)).

#### 3.3.3. Apoptosis detection by EB/AO differential staining

**Figure 4.**
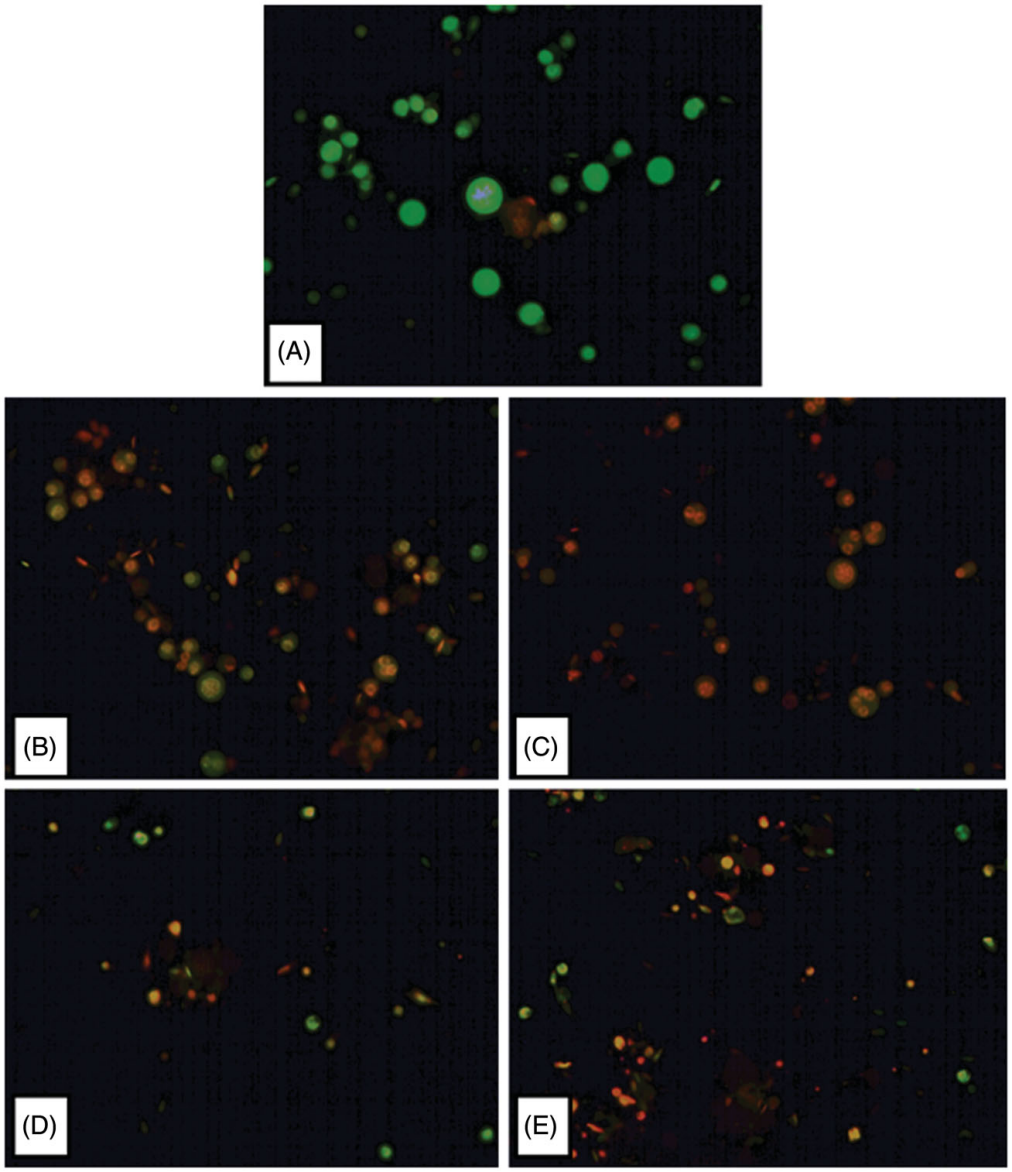
Fluorescent microphotographs of cells post treatment with different compounds (B–E) at 10 μM concentration for 6 h duration in comparison to the control (A). Viable healthy cells appeared bright green, while apoptotic cells appeared orange-red (EB/AO stain, 400×).

The correlation between reduced cell viability and apoptosis induction in testicular cells was determined by EB/AO differential staining method. Here, cells that exhibited bright green fluorescence were marked as healthy normal cells and cells that exhibited orange-red fluorescence were marked as apoptotic dead cells. Figure 4(B–E) has clearly demonstrated the potency of given compounds as apoptotic inducers as more number of cells with orange-red fluorescence were present in treated groups as compared to the control group (Figure 4(A)).

**Figure 5.**
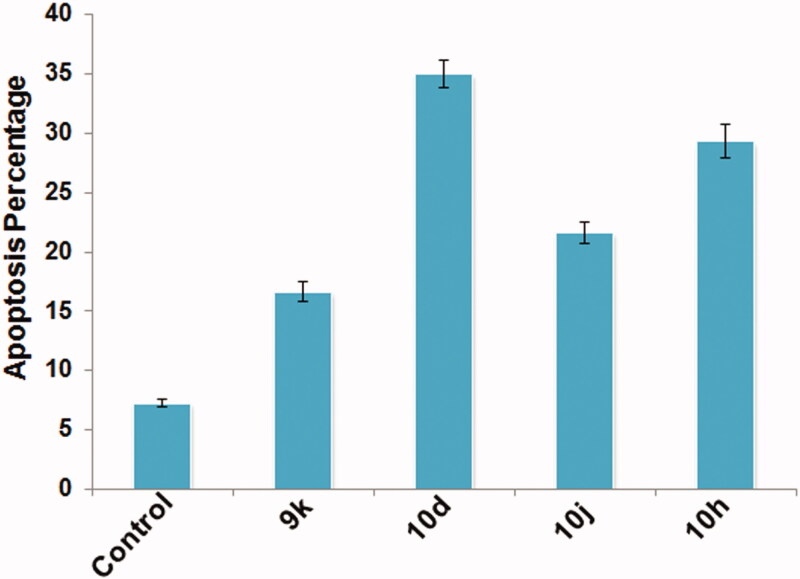
Graph depicting percent apoptosis in testicular cells post treatment with different compounds at 10 μM concentration for 6 h duration. Data are expressed as mean ± SEM using statistical software SPSS 16.

Therefore, all the four given compounds induced apoptosis in testicular cells, suggesting their cyto-genotoxic nature. However, the quantification of apoptosis revealed that higher percentage of apoptosis in testicular cells was noticed in groups treated with compounds **10d** and **10h** with values 35.00 ± 1.15 and 29.33 ± 1.45, respectively (Figure 5). Similar increasing trend was observed in other treated groups as compared to the control group (7.33 ± 0.33).

#### 3.3.4. Oxidative stress measurements

**Figure 6.**
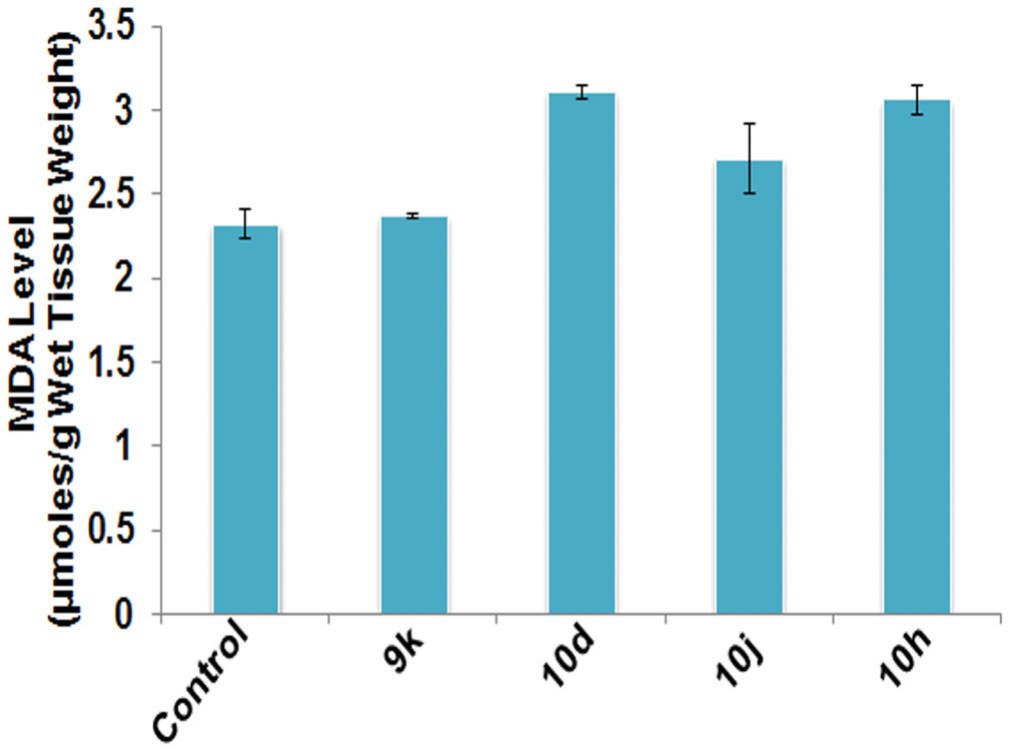
Graph depicting MDA level in testicular tissue post treatment with different compounds at 10 μM concentration for 6 h duration. Data are expressed as mean ± SEM using statistical software SPSS 16.

**Figure 7.**
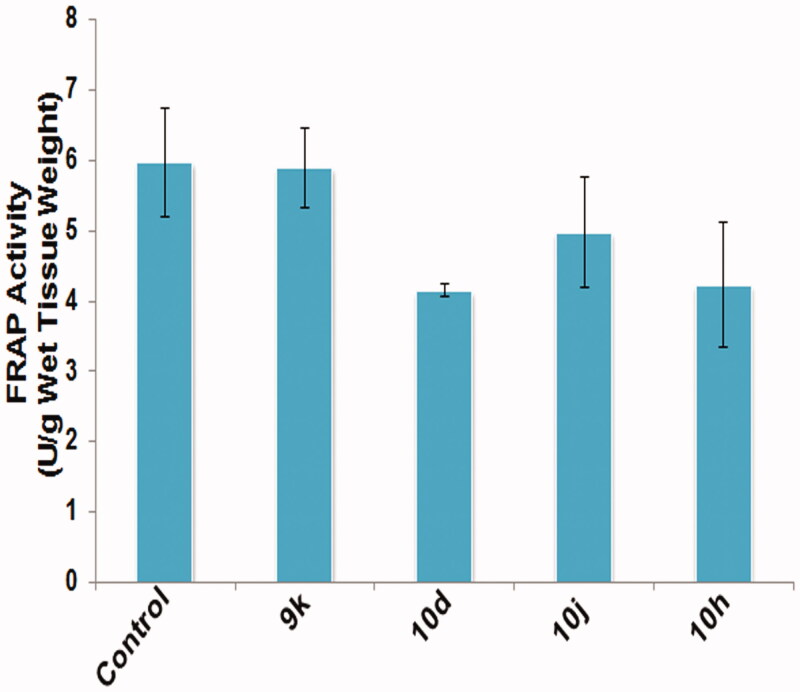
Graph depicting FRAP activity in testicular tissue post treatment with different compounds at 10 μM concentration for 6 h duration. Data are expressed as mean ± SEM using statistical software SPSS 16.

Oxidative stress has been associated with apoptosis-mediated cytotoxicity. Level of MDA and total antioxidant capacity (FRAP activity) were used as markers of oxidative stress in testicular tissue. All the groups treated with different compounds have shown elevated levels of MDA in testicular tissue (Figure 6) in comparison to the control group. In contrast, the FRAP activity has declined in groups treated with the given compounds as compared to the control group, which indicates reduced antioxidant capacity of testicular tissue (Figure 7). Therefore, increased MDA level and decreased antioxidant capacity induced oxidative stress in testicular tissue which in turn causes apoptosis of testicular cells.

## 4. Conclusion

Herein we report the synthesis and biological evaluation of two subsets possessing 1,2,3-triazole appended four- and three-substituted benzenesulphonamides **9–10**. All the target compounds were assayed *in vitro* for the CA inhibition studies against ubiquitous hCA isoforms I and II, glaucoma-associated hCA IV and tumour-associated isoform hCA IX. Potent and selective compounds from CA inhibition studies were also investigated for apoptosis induction in mammalian cells. The hCA inhibition profile displayed that the target compounds were moderate to weak inhibitors of hCA I and IV isoforms in reference to the standard drug acetazolamide. While in case of hCA II, most of the four-substituted benzenesulphonamide derivatives exhibited low nanomolar inhibition and phenyl substituted derivative **9k** was the least effective inhibitor with K_I_ value 833.3 nM. All the target compounds showed excellent inhibition profile for hCA IX with K_I_ values ranging from 16.4 to 66.0 nM and chloro-substituted compound **10d** was the most potent inhibitor as compared to the standard drug acetazolamide. The selectivity index revealed compounds **9h** and **10h** as the most selective compounds for hCA IX over ubiquitous isoform hCA I and compounds **9k** and **10j** as the most selective compounds for hCA IX over hCA II in respective subsets. The ability of the four target compounds **9k, 10d, 10h,** and **10j** to induce apoptosis was assessed *in vitro* with the help of apoptosis quantification studies along with other assays examined in goat testicular cells. MTT assay was performed to assess the cytotoxicity of the target compounds and the results exhibited significant reduction in testicular cell viability with maximum relative decrease observed with compounds **10d** and **10h** having a halogen substituent. Oxidative stress caused by the target compounds in treated tissues was investigated *via* TBARS and FRAP assays which revealed that the tested compounds induced oxidative stress in testicular tissue leading to apoptosis of testicular cells. In addition, histopathological alterations and EB/AO differential staining demonstrated the apoptosis induction potential of the selected target compounds in goat testicular cells with compounds **10d** and **10h**, both having a halogen substituent, exhibiting higher percentage of apoptosis amongst the tested compounds. Among all the considered compounds in the present study, **10d** and **10h** are the most relevant compounds possessing interesting selective hCA IX inhibition and apoptotic induction effects. Considering the scarcity of data available about the apoptosis induction potential of isoform-selective hCA inhibitors, present study helps in bridging this important gap that have the potential to contribute in the design and development of hCA inhibitors capable of inducing apoptosis as highly promising anticancer agents.

## Supplementary Material

Supplemental Material
