# Supplementary material for: Selective inhibition of carbonic anhydrase IX by sulphonylated 1,2,3-triazole incorporated benzenesulphonamides capable of inducing apoptosis
Source: J Enzyme Inhib Med Chem. 2022 May 26;37(1):1454–63. doi: 10.1080/14756366.2022.2077333 (PMC9154810; doi:10.1080/14756366.2022.2077333)

Supplementary Information

Selective inhibition of carbonic anhydrase IX by sulfonylated 1,2,3-triazole incorporated benzenesulfonamides capable of inducing apoptosis

Kiran Siwach[a], Amit Kumar[a], Harish Panchal[b], Rajiv Kumar[c], Jitender Kumar Bhardwaj[b], Andrea Angeli,[d] Claudiu T. Supuran[d]*, Pawan K. Sharma[a]*

[a]Department of Chemistry, Kurukshetra University, Kurukshetra, Haryana, 136119, India.

[b]Reproductive Physiology Laboratory, Department of Zoology, Kurukshetra University, Kurukshetra, Haryana, 136119, India.

[c]Ch. Mani Ram Godara Government College for Women, Bhodia Khera, Fatehabad, Haryana, 125050, India.

[d] Department of Neurosciences, Psychology, Drug Research and Child Health, Pharmaceutical and Nutraceutical Section, University of Florence, Florence, Italy.

*Corresponding authors: Tel.: +91 9416457355; Fax: +91 1744 238277; e-mail: pksharma@kuk.ac.in (PKS); Tel/Fax: +39-055-4573005, e-mail: claudiu.supuran@unifi.it (CTS)

Contents

^{1}H and ^{13}C NMR Spectra of Compounds: **9a-9k** and **10a-10k.**

(9a) ^{1}H NMR

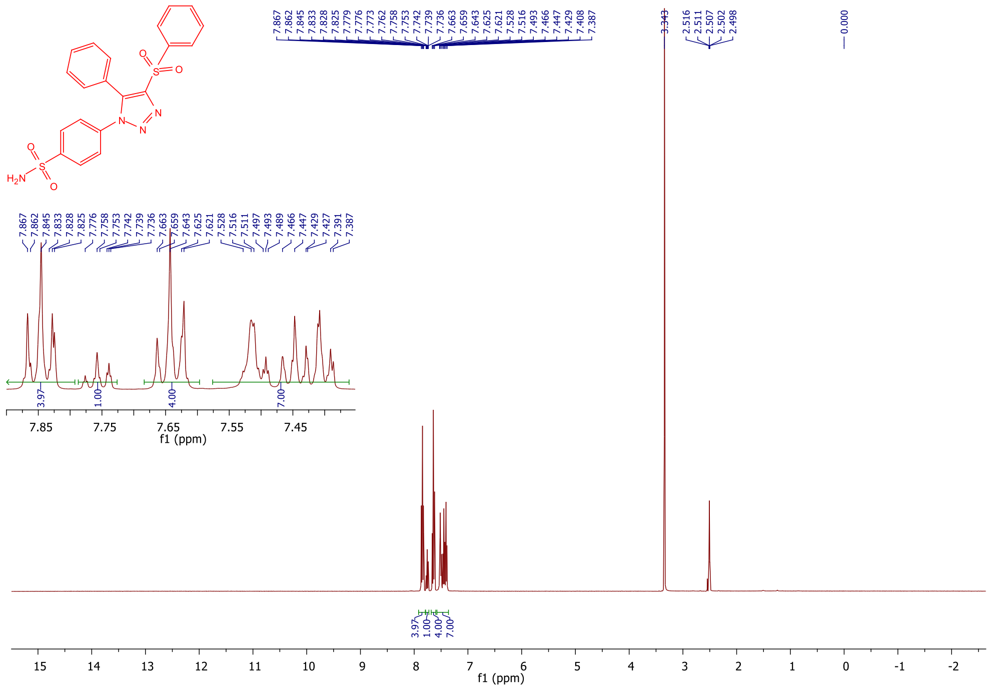

(9a) ^{13}C NMR

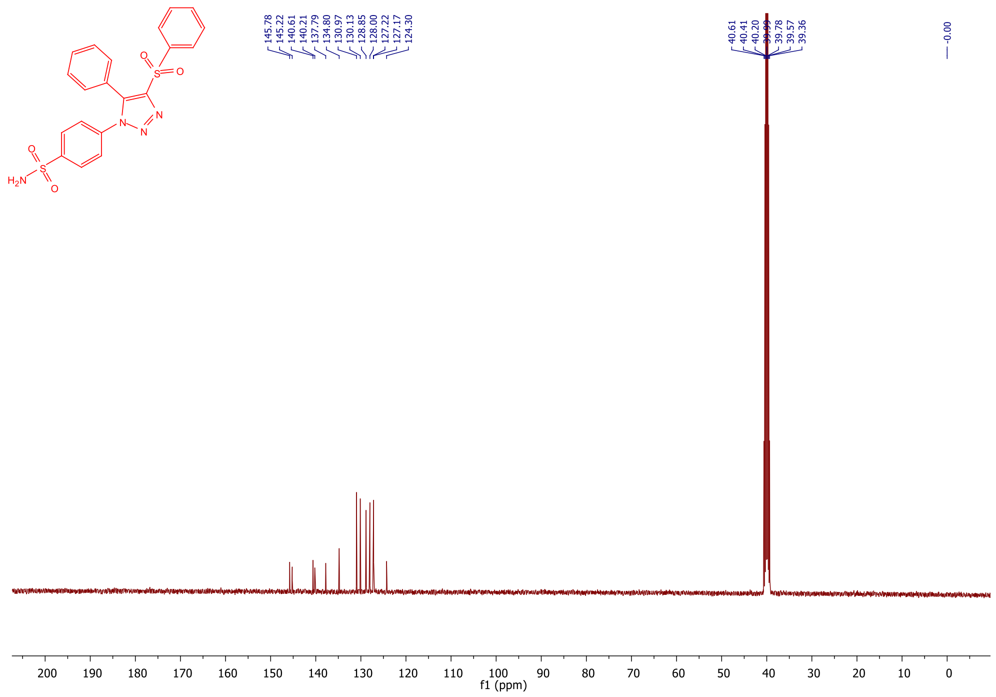

(9b) ^{1}H NMR

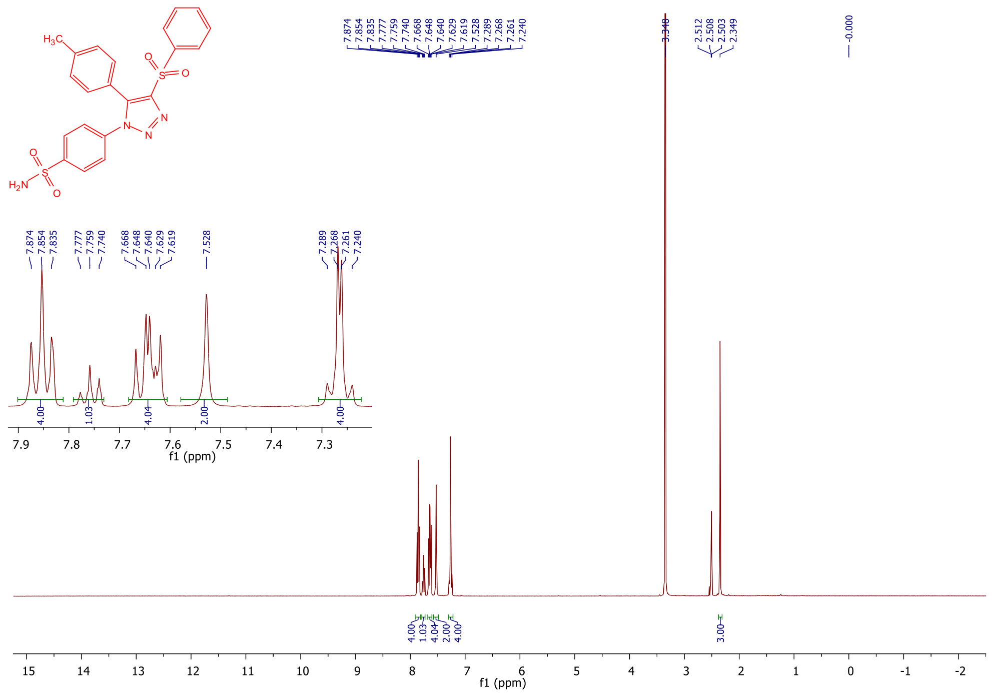

(9b) ^{13}C NMR

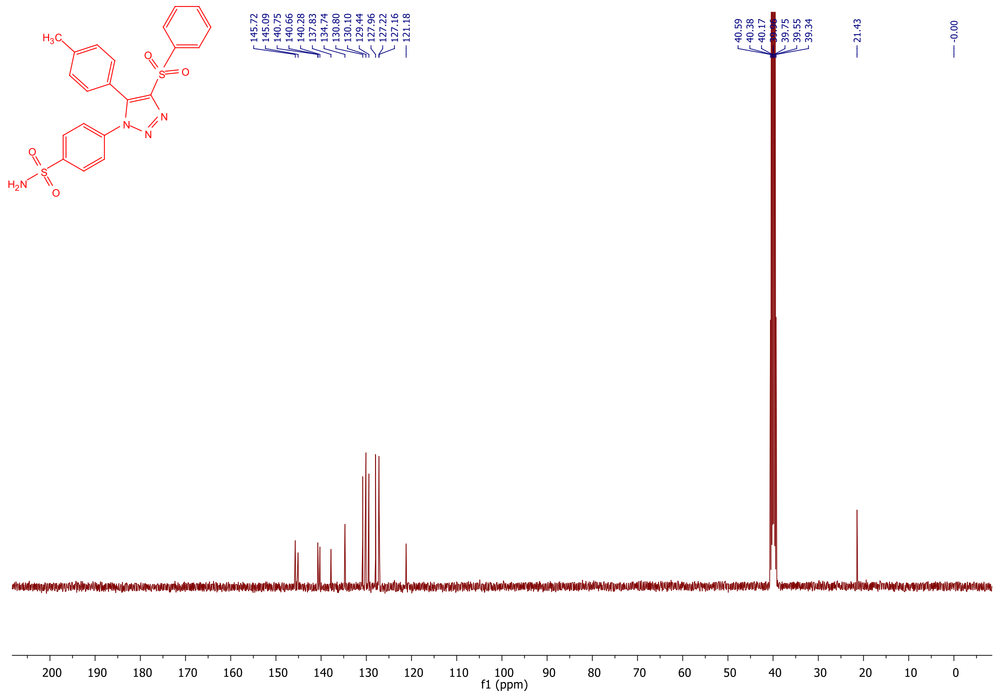

(9c) ^{1}H NMR

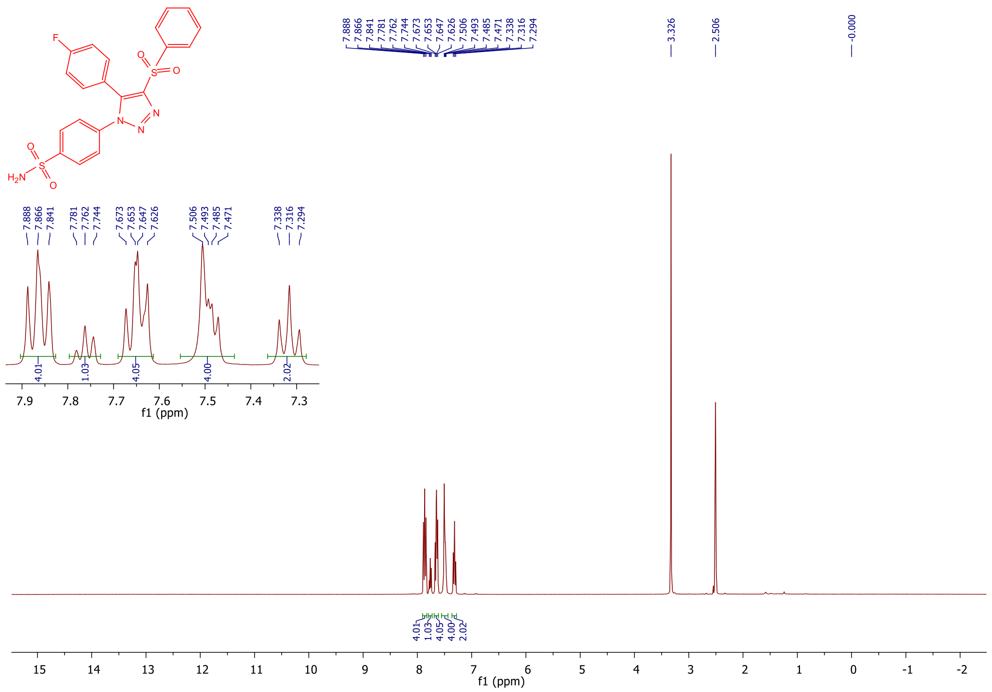

(9c) ^{13}C NMR

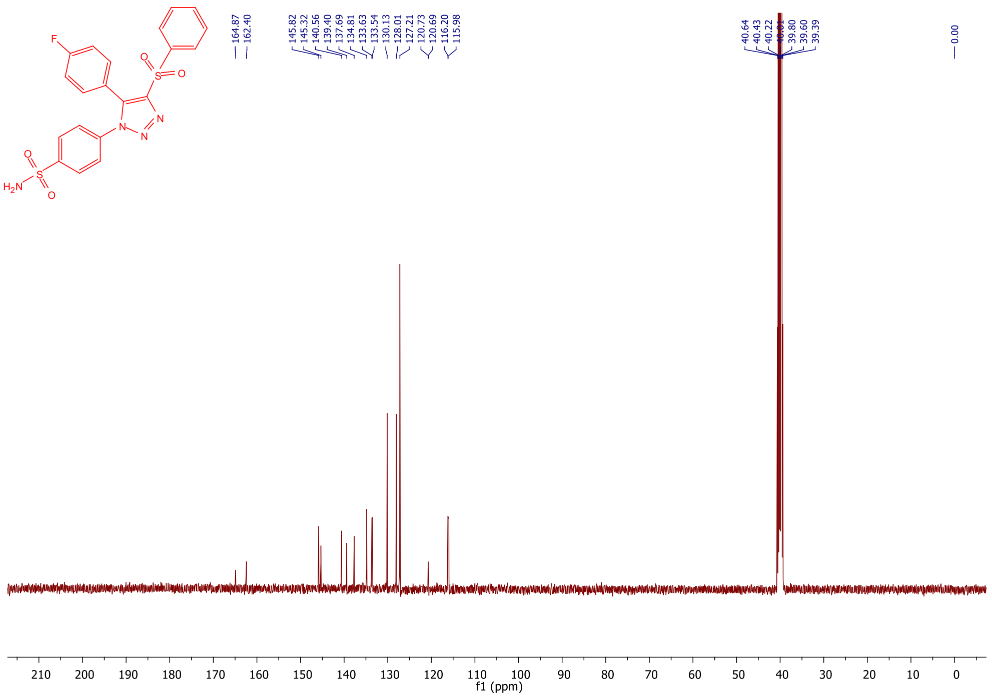

(9d) ^{1}H NMR

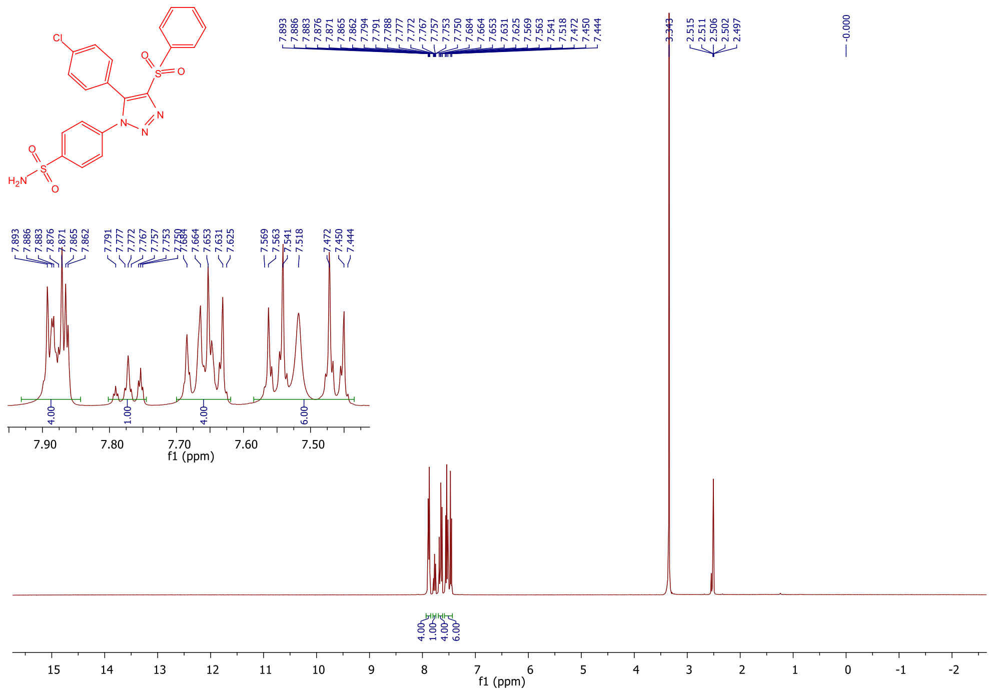

(9d) ^{13}C NMR

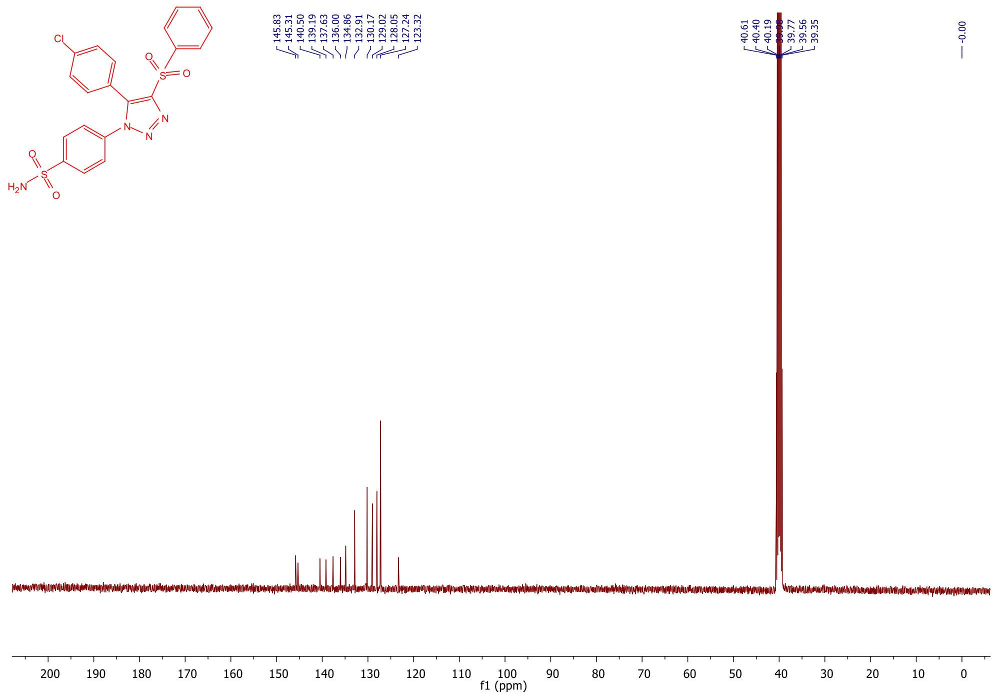

(9e) ^{1}H NMR

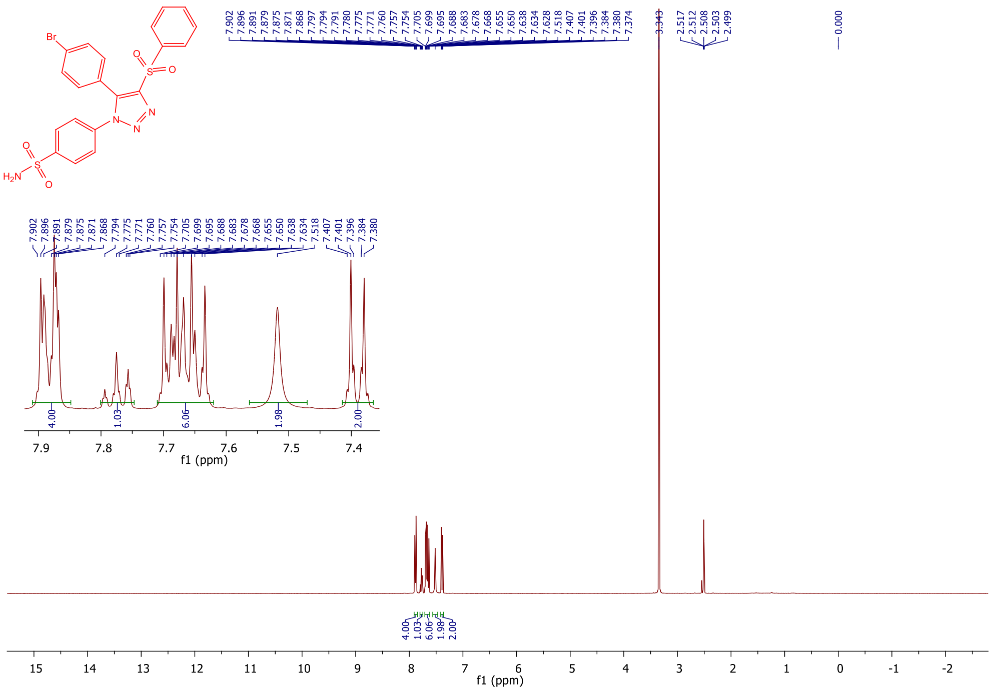

(9e) ^{13}C NMR

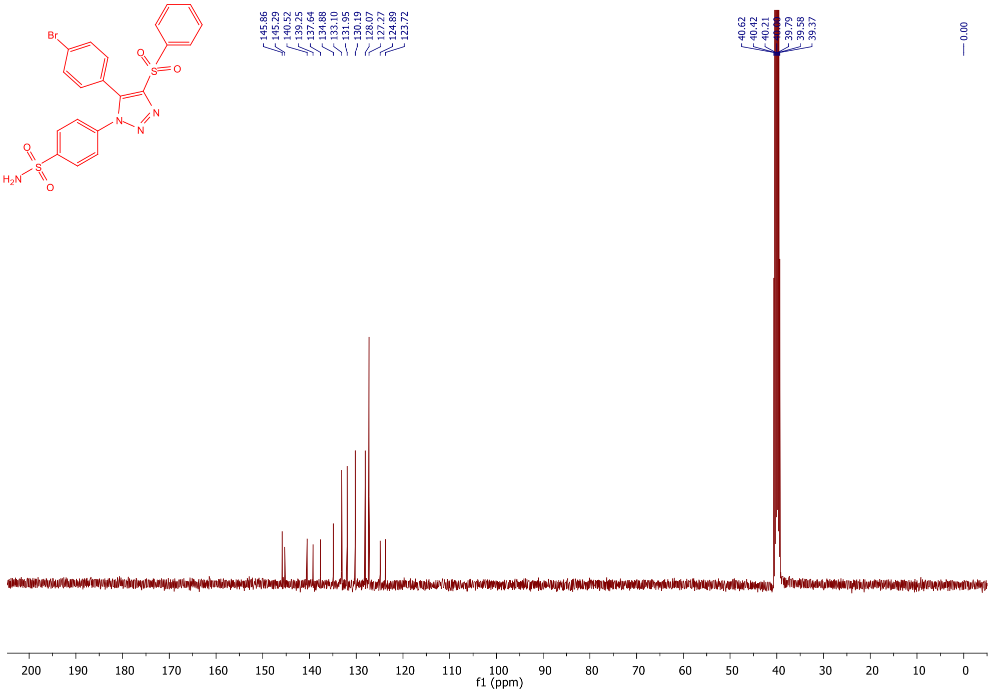

(9f) ^{1}H NMR

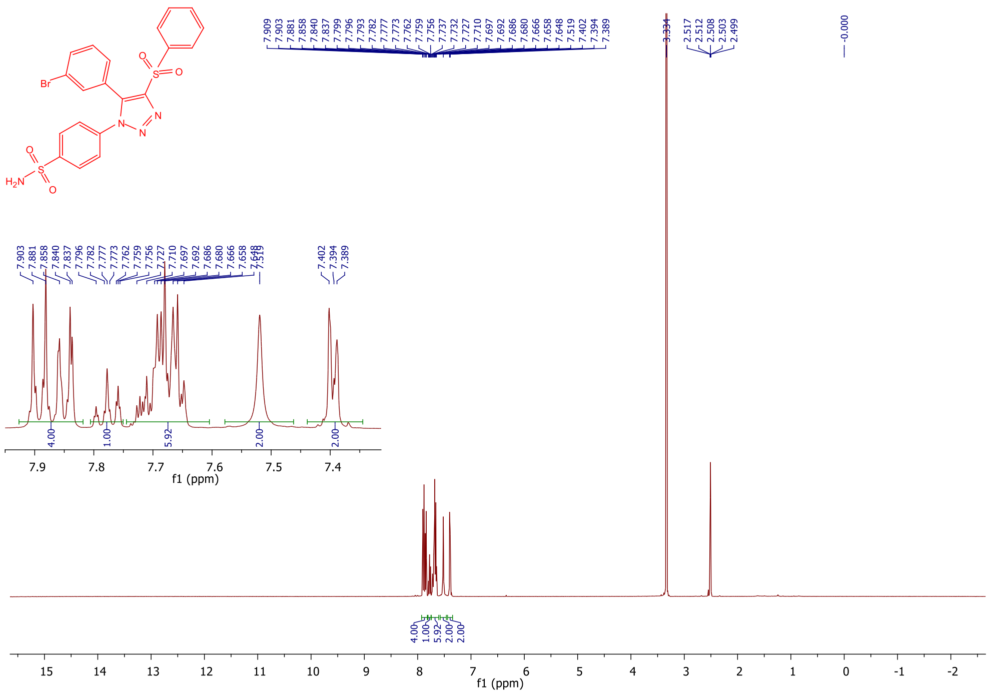

(9f) ^{13}C NMR

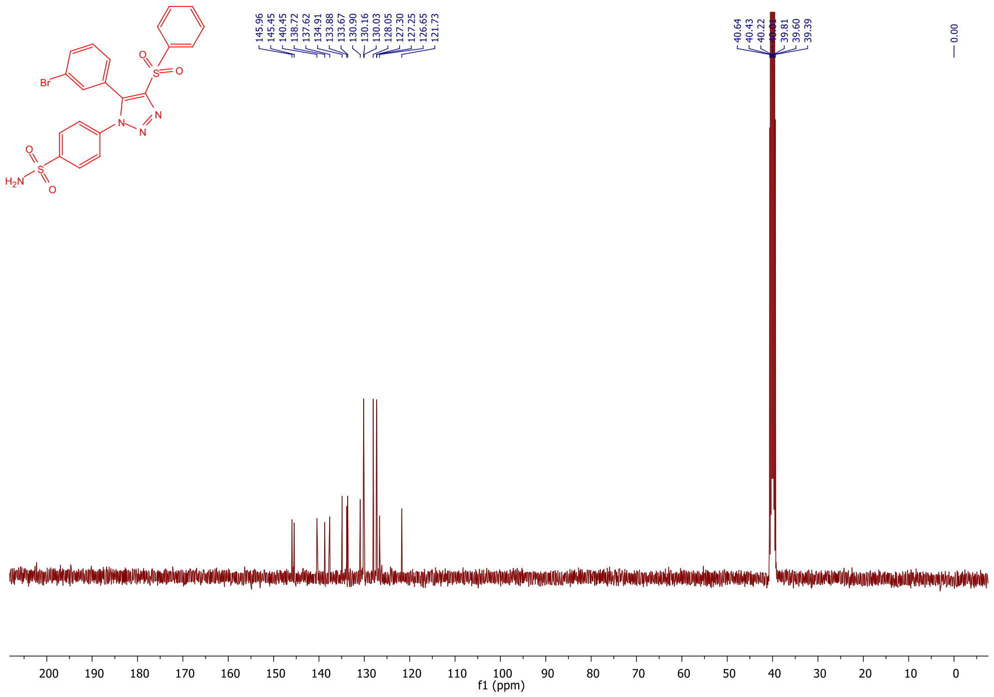

(9g) ^{1}H NMR

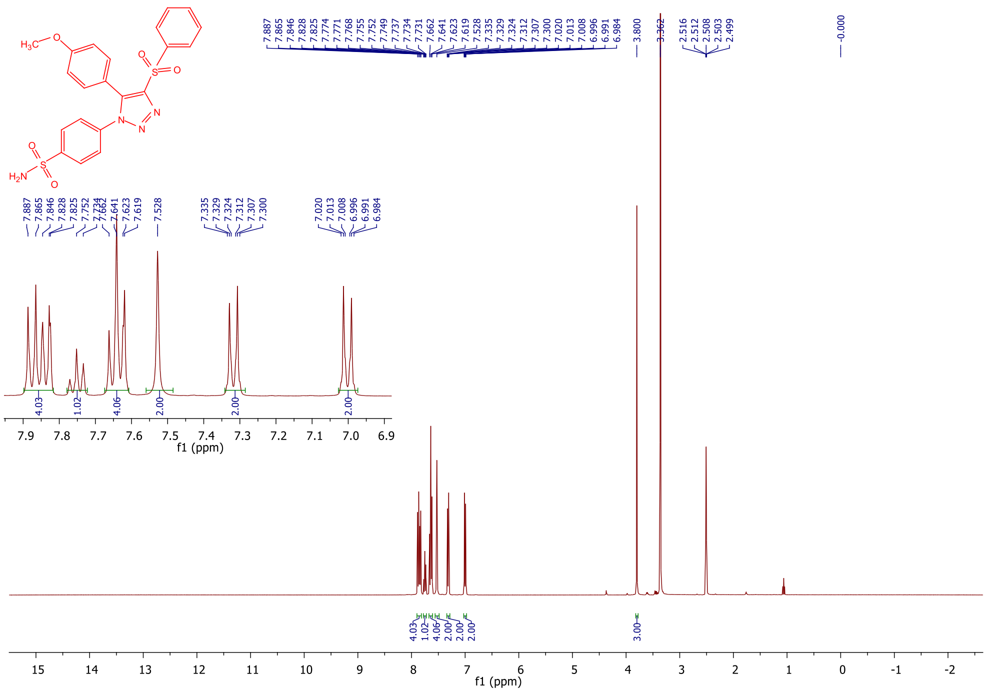

(9g) ^{13}C NMR

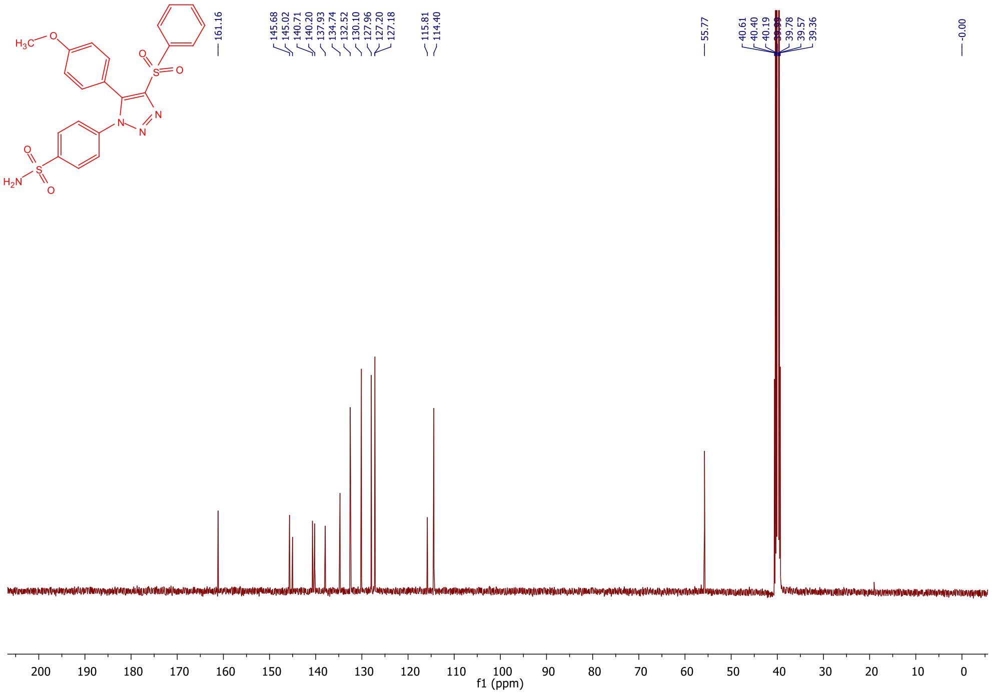

(9h) ^{1}H NMR

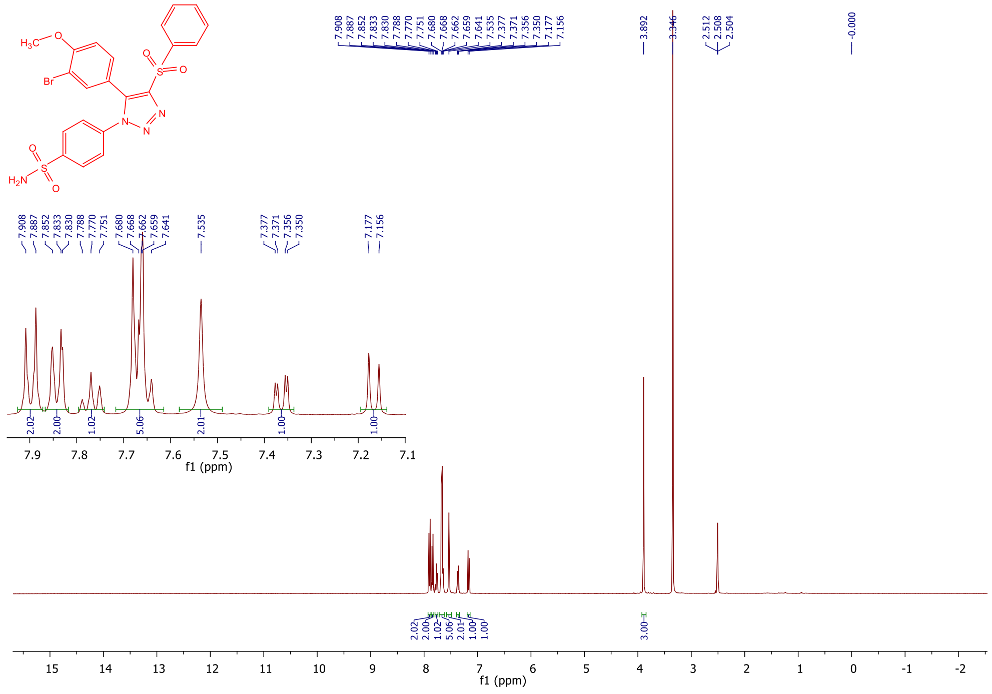

(9h) ^{13}C NMR

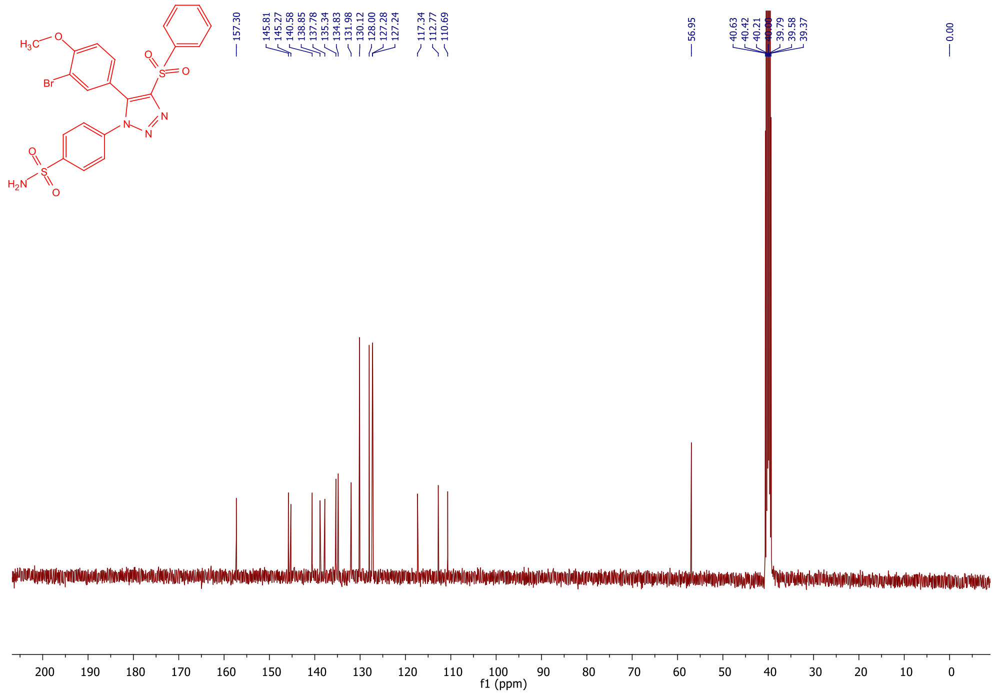

(9i) ^{1}H NMR

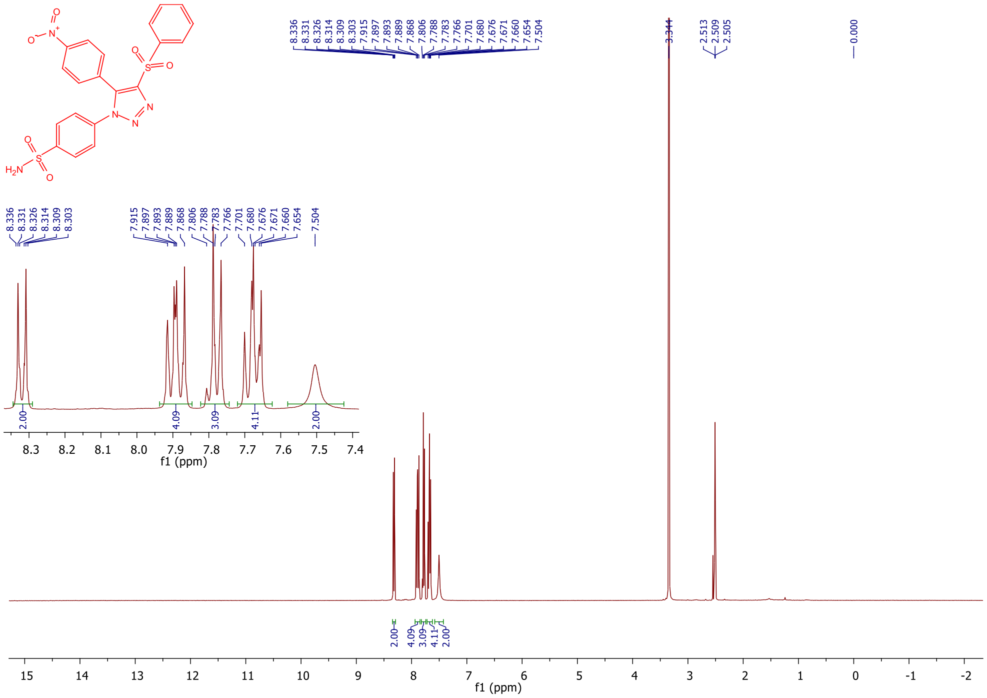

(9i) ^{13}C NMR

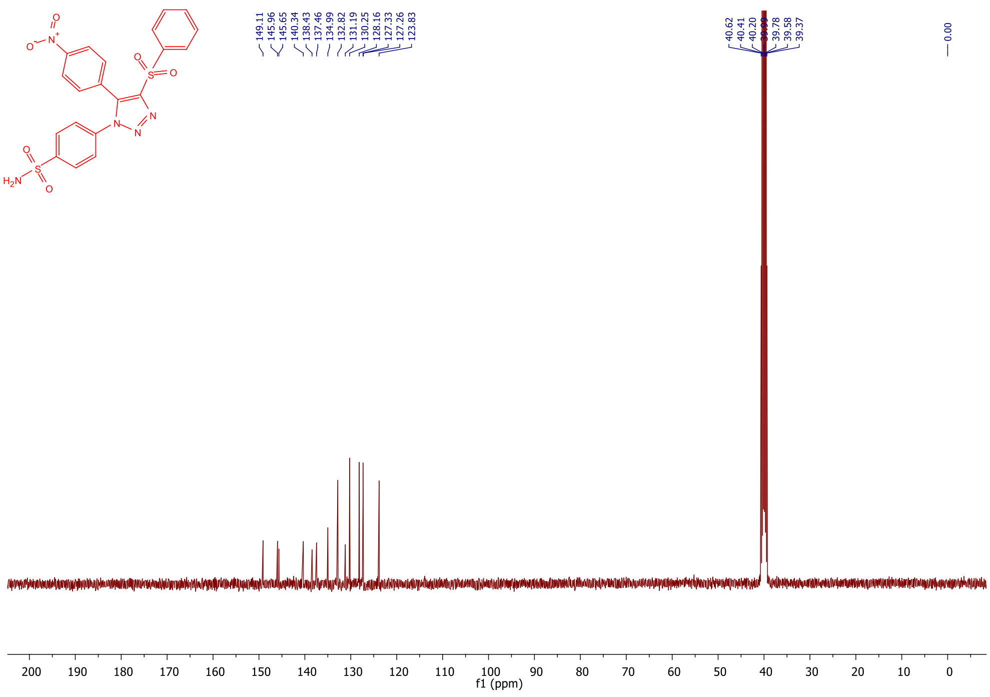

(9j) ^{1}H NMR

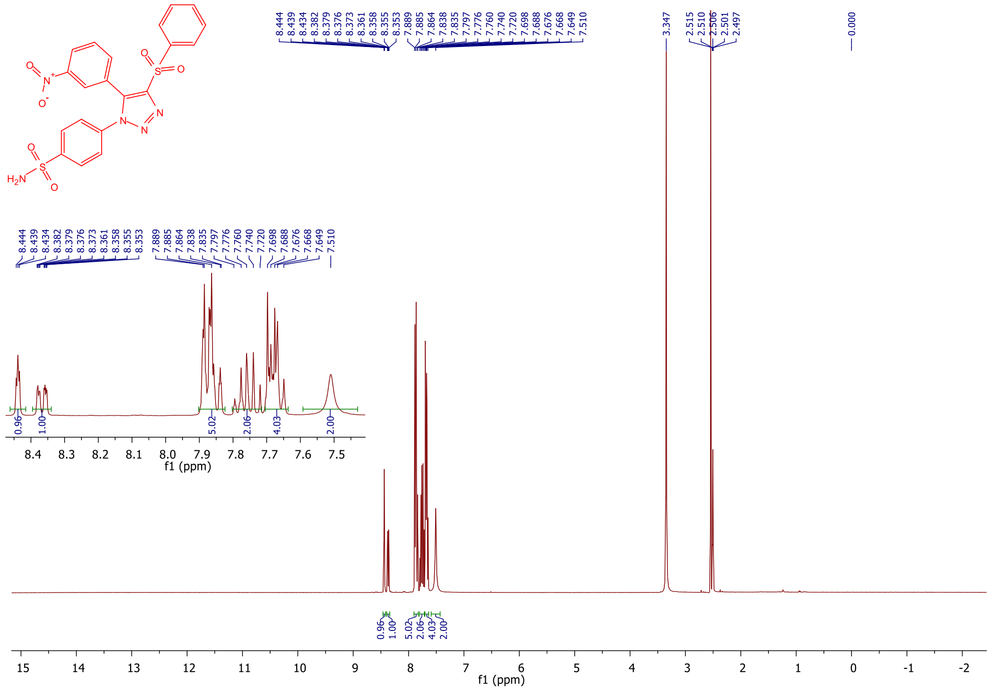

(9j) ^{13}C NMR

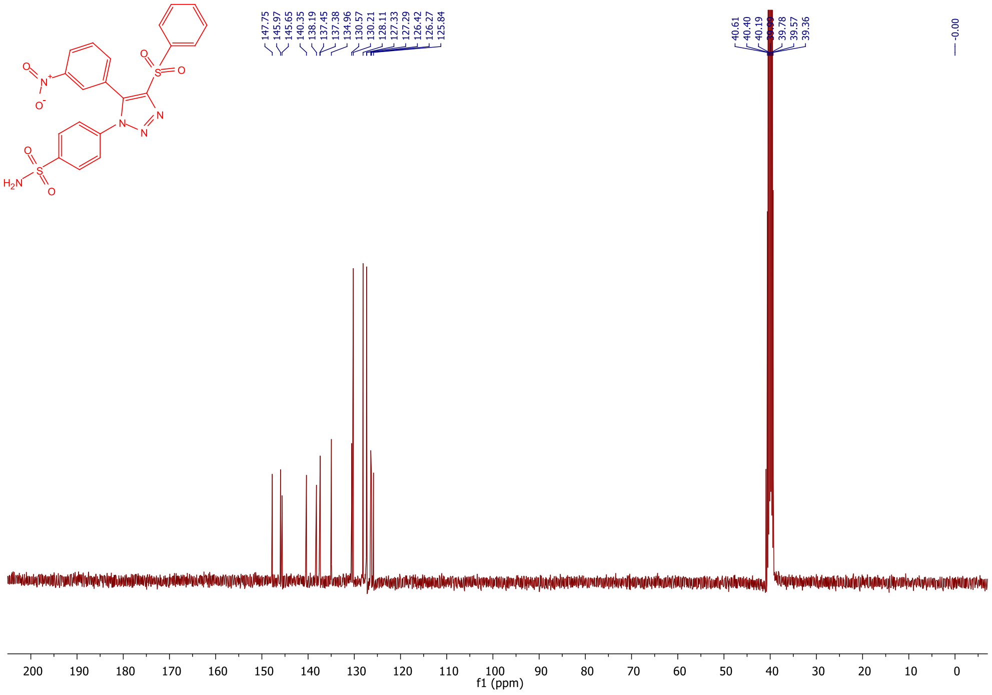

(9k) ^{1}H NMR

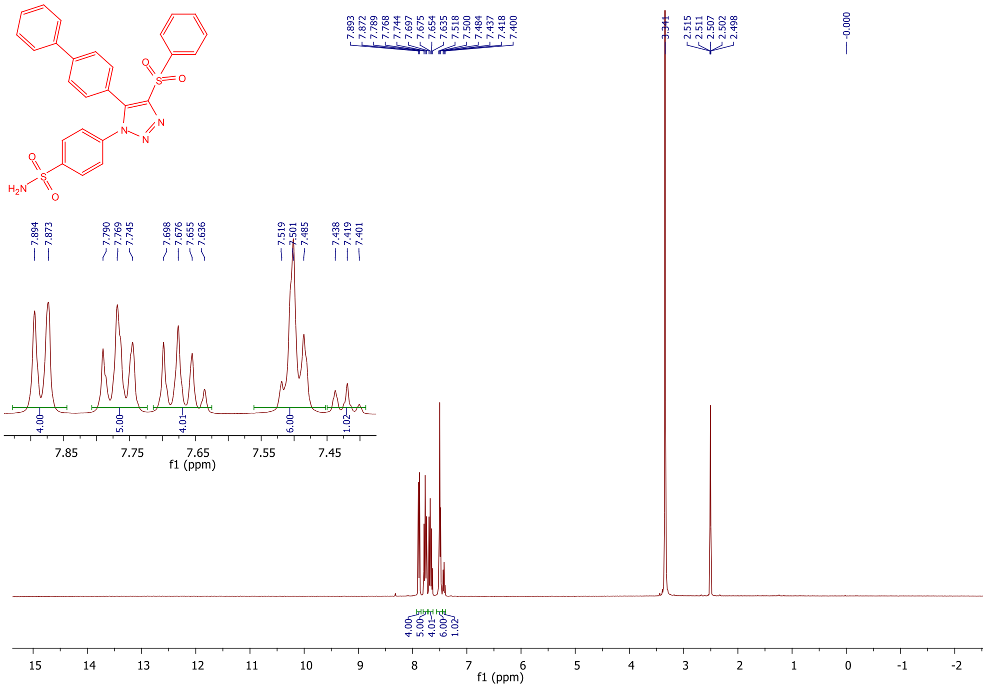

(9k) ^{13}C NMR

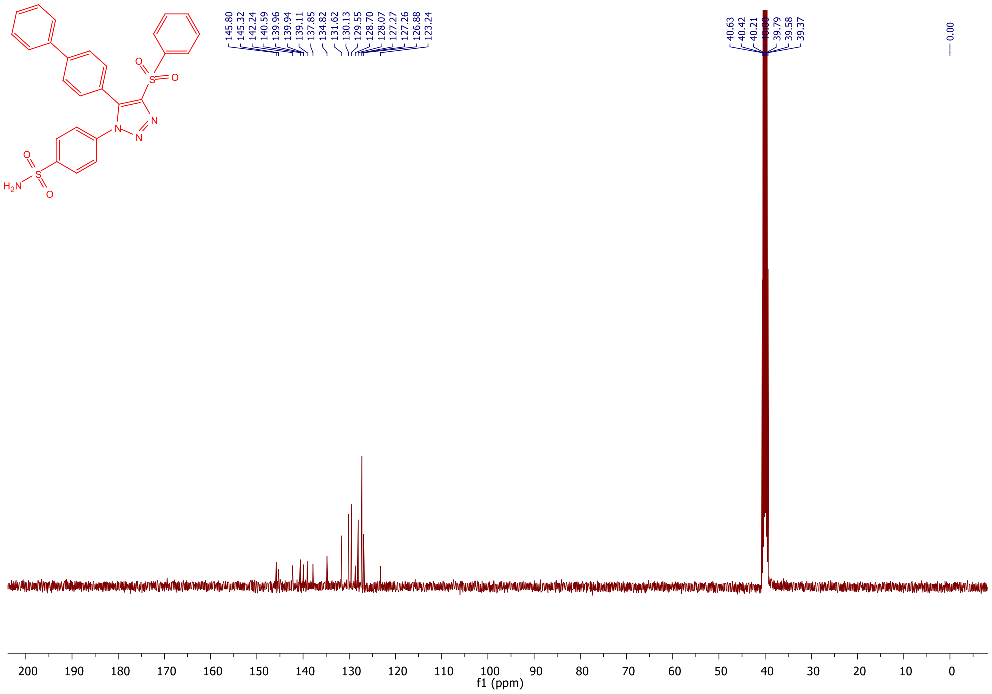

(10a) ^{1}H NMR

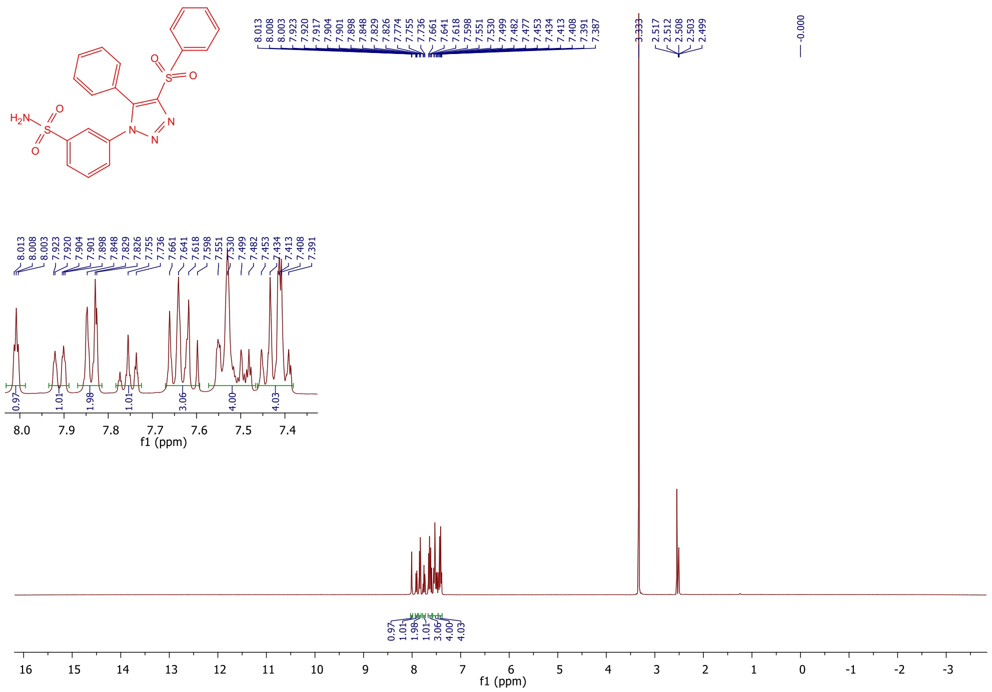

(10a) ^{13}C NMR

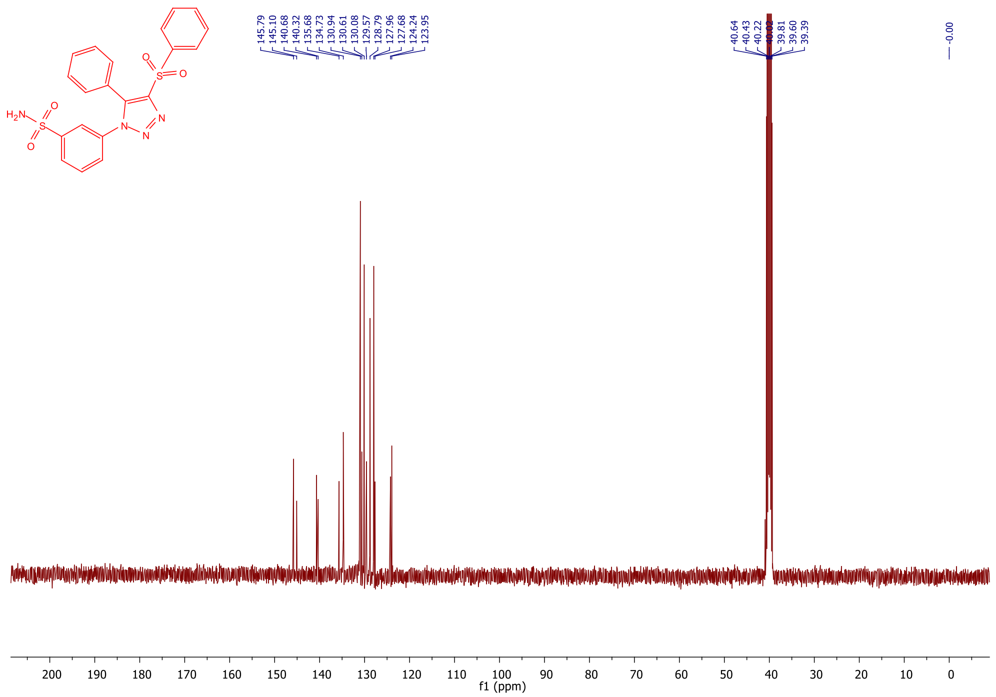

(10b) ^{1}H NMR

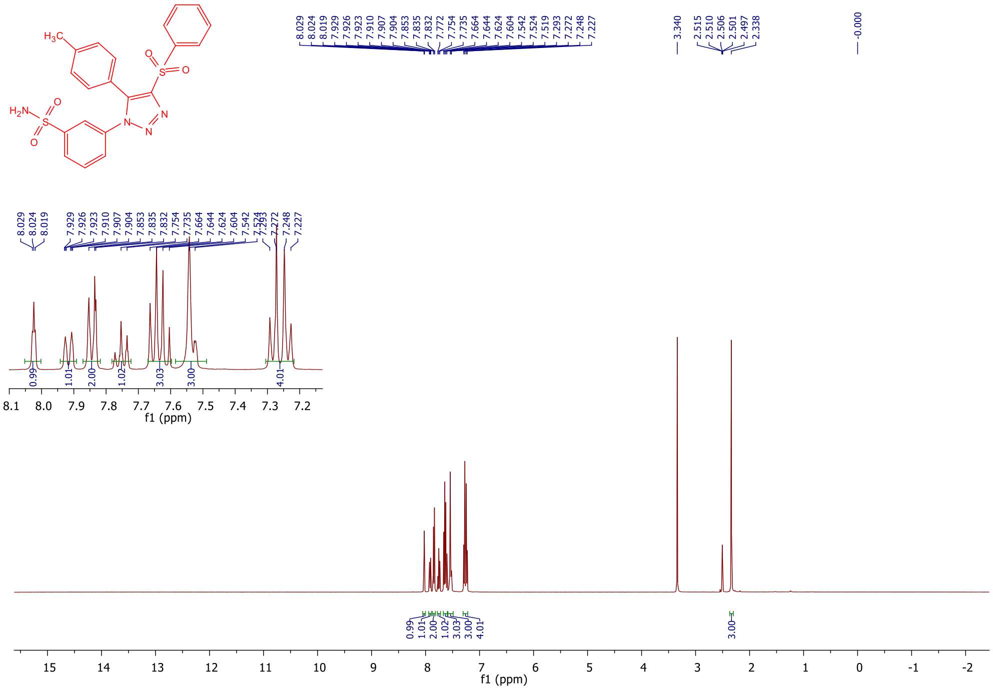

(10b) ^{13}C NMR

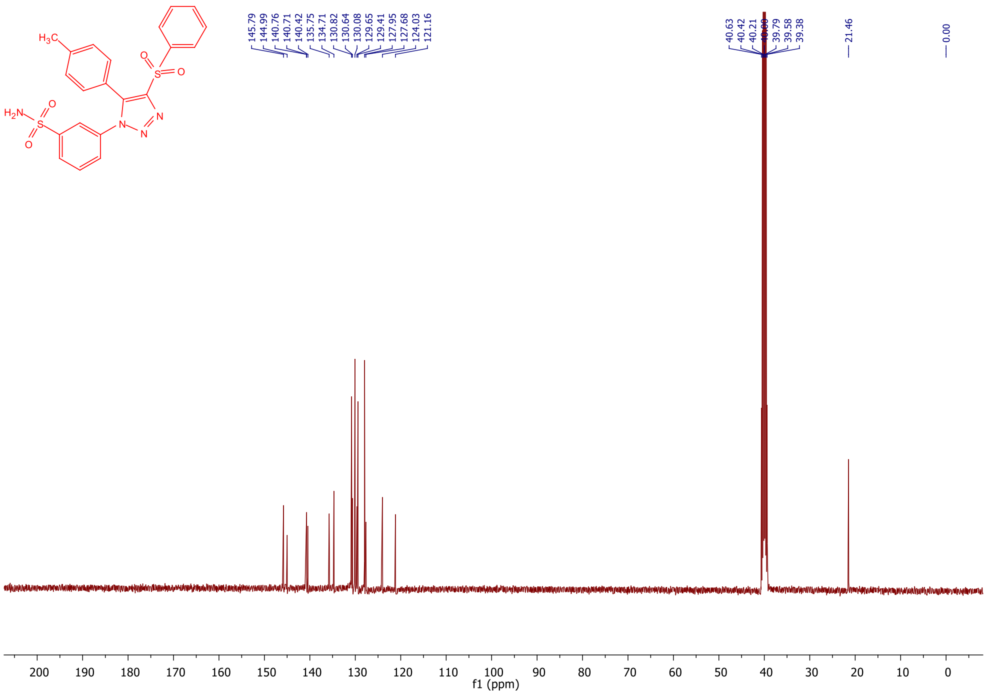

(10c) ^{1}H NMR

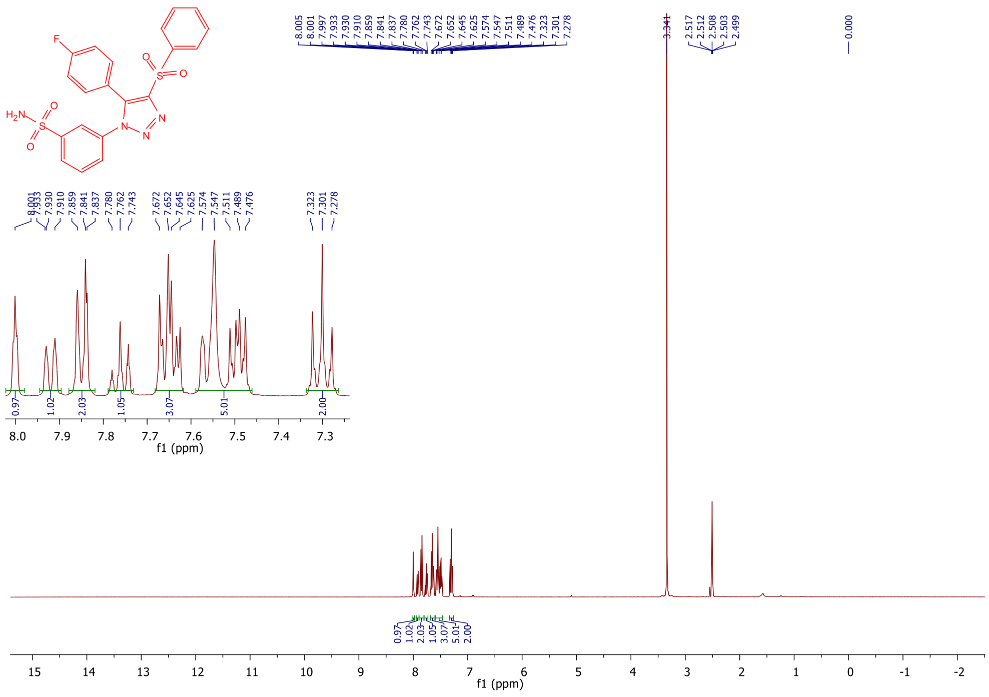

(10c) ^{13}C NMR

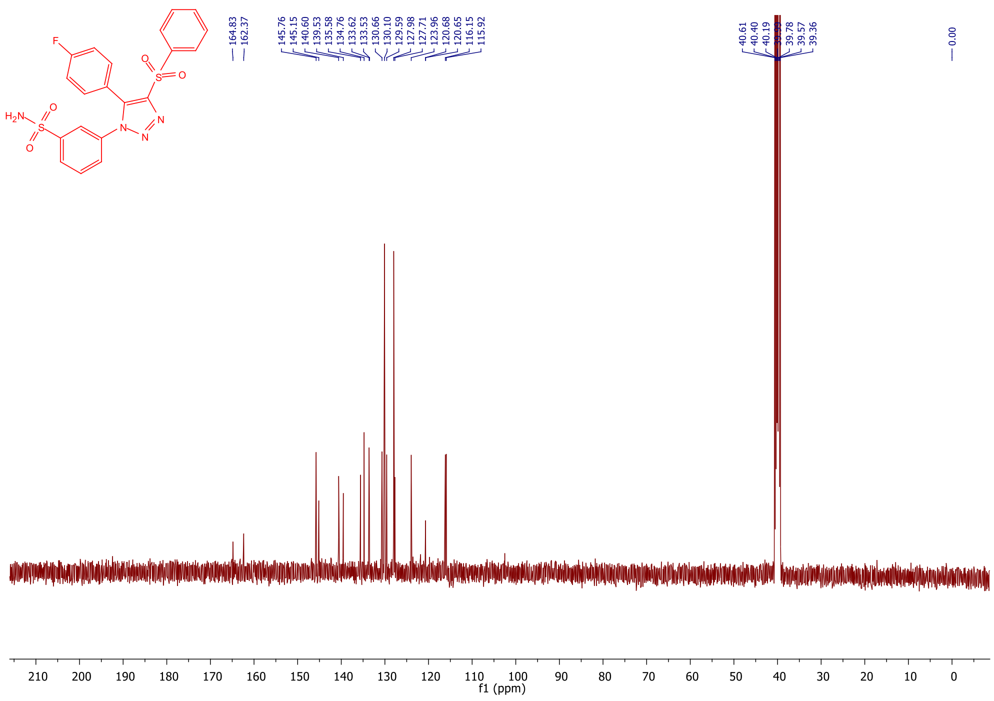

(10d) ^{1}H NMR

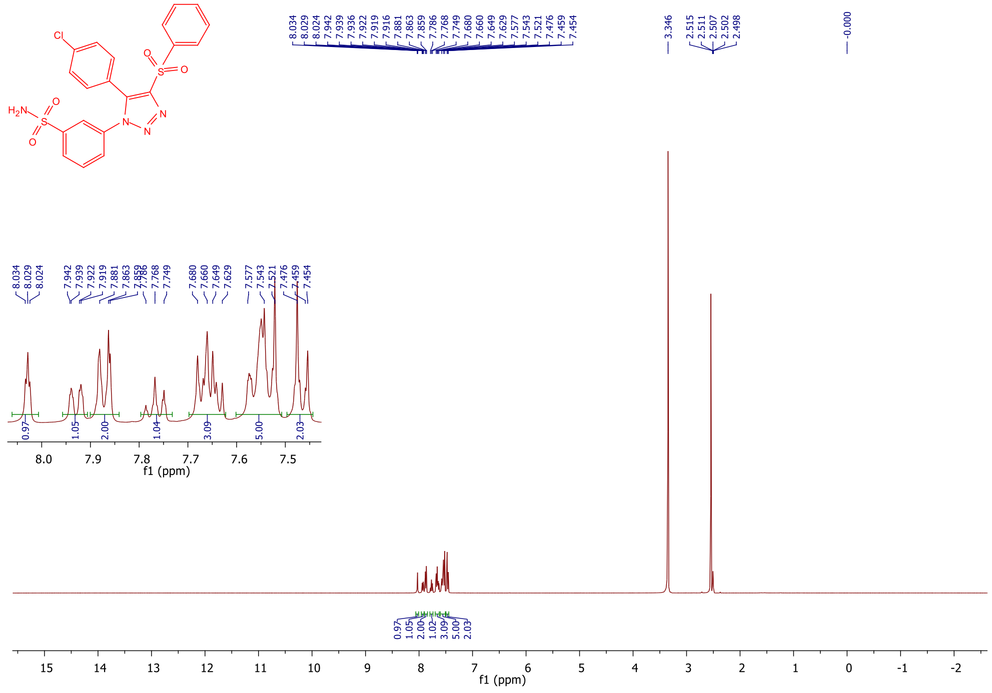

(10d) ^{13}C NMR

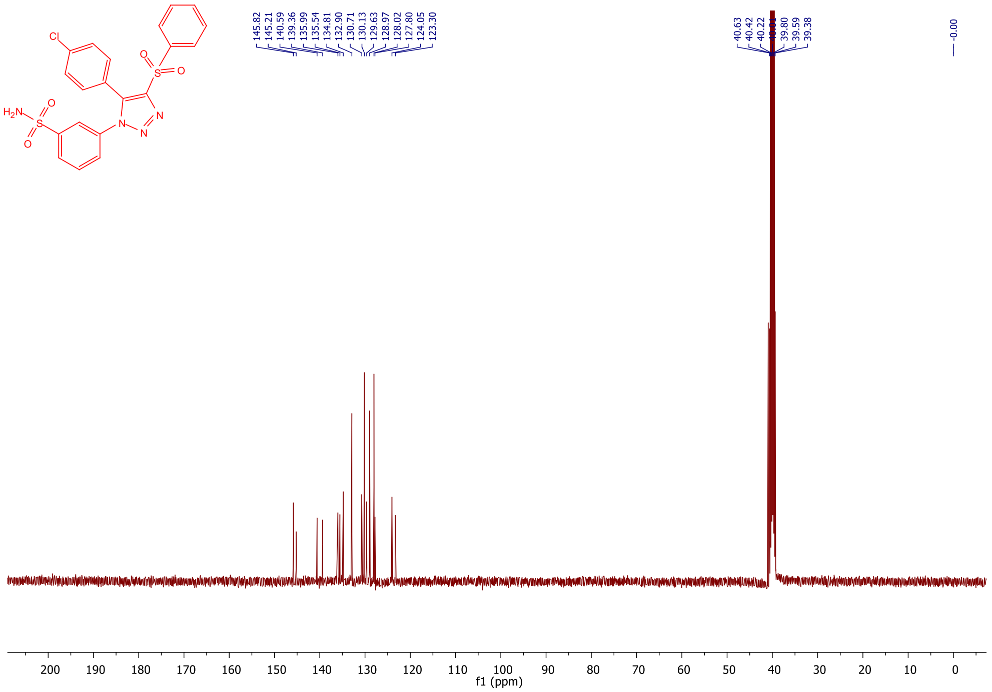

(10e) ^{1}H NMR

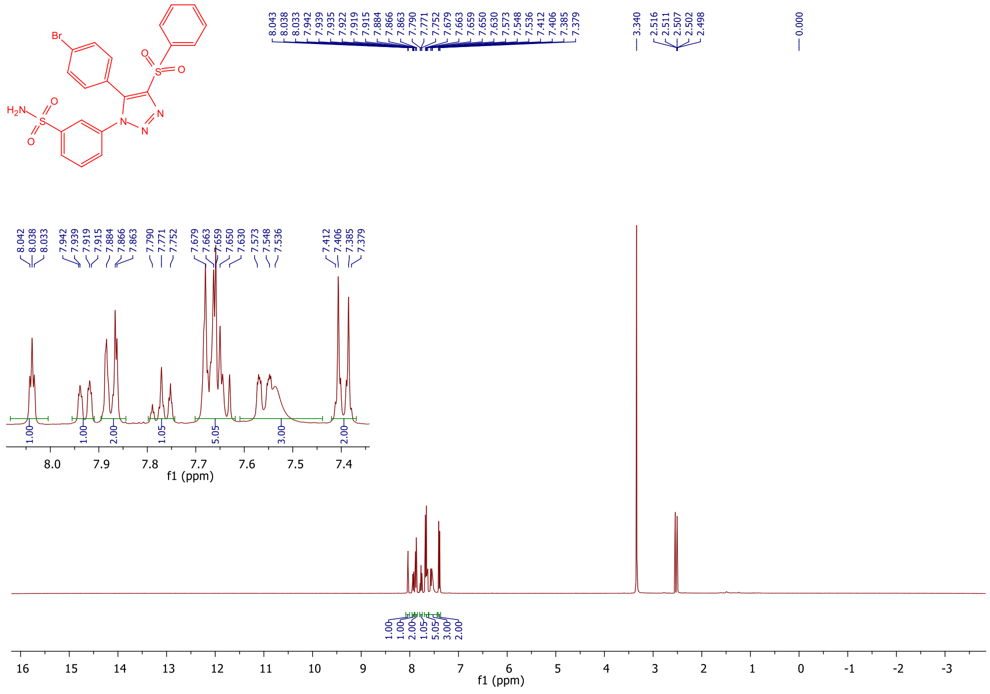

(10e) ^{13}C NMR

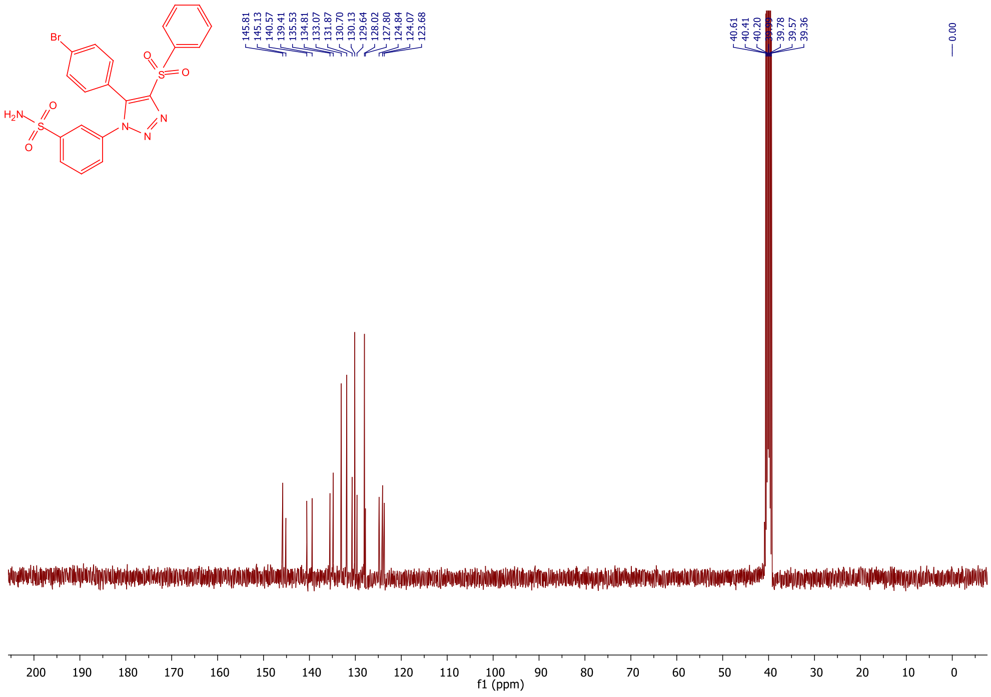

(10f) ^{1}H NMR

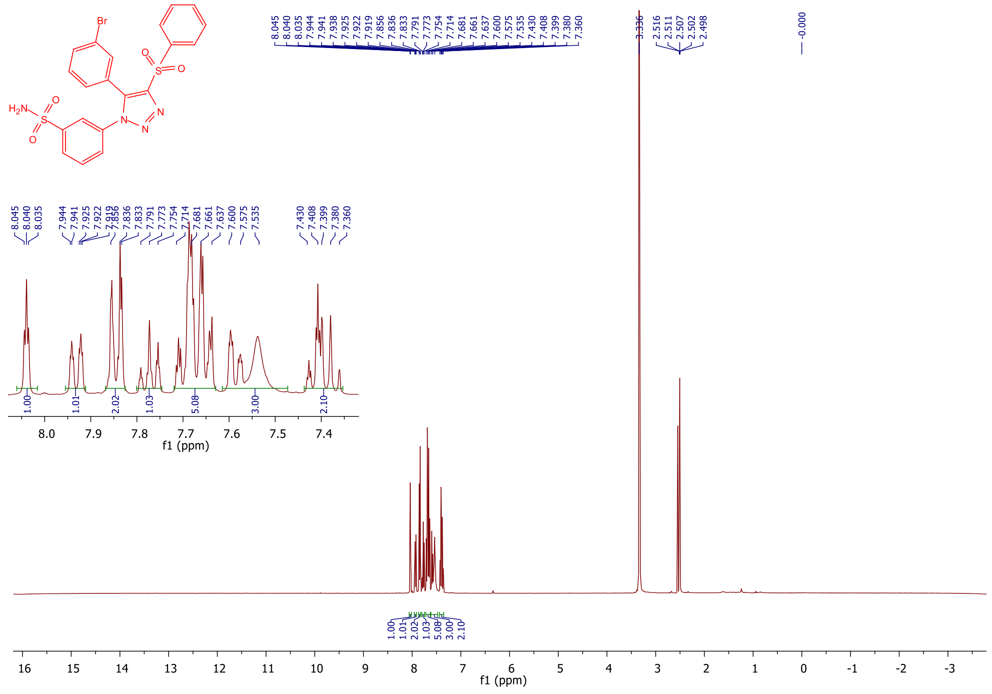

(10f) ^{13}C NMR

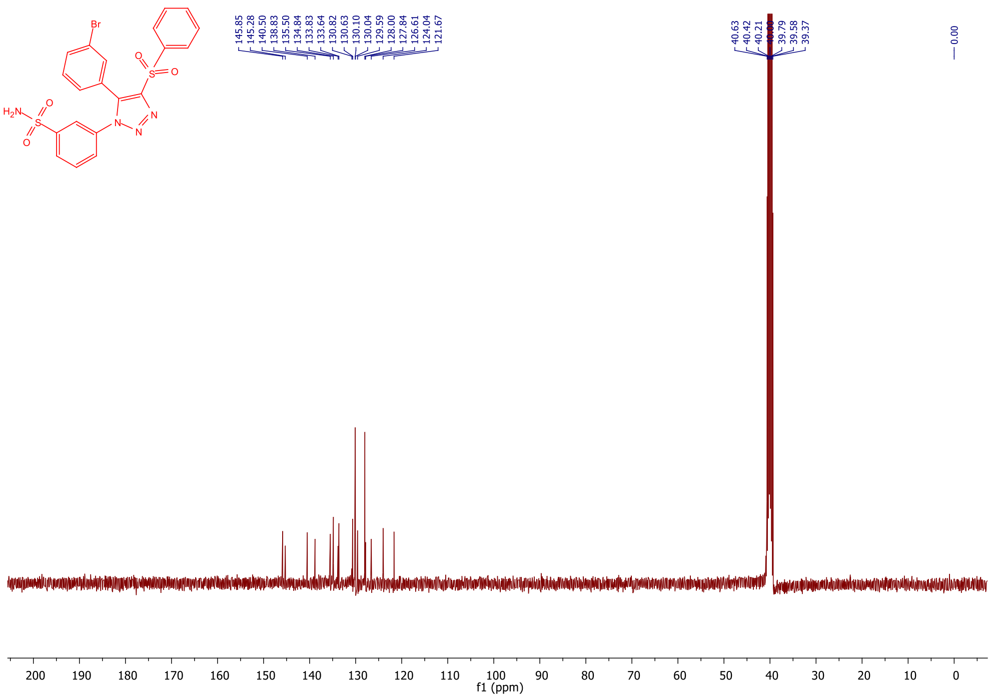

(10g) ^{1}H NMR

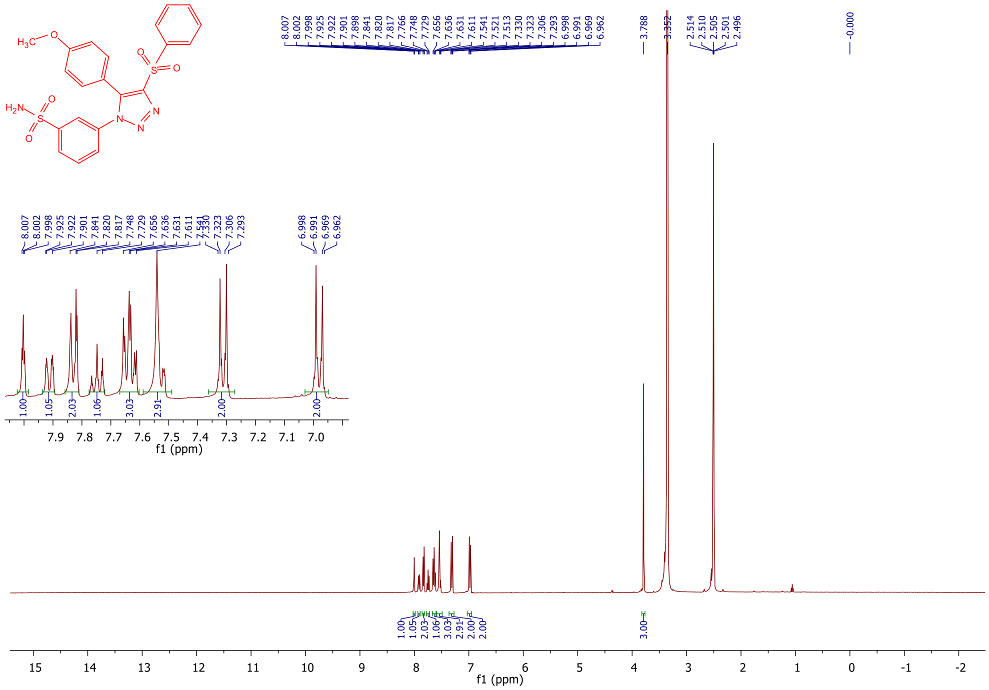

(10g) ^{13}C NMR

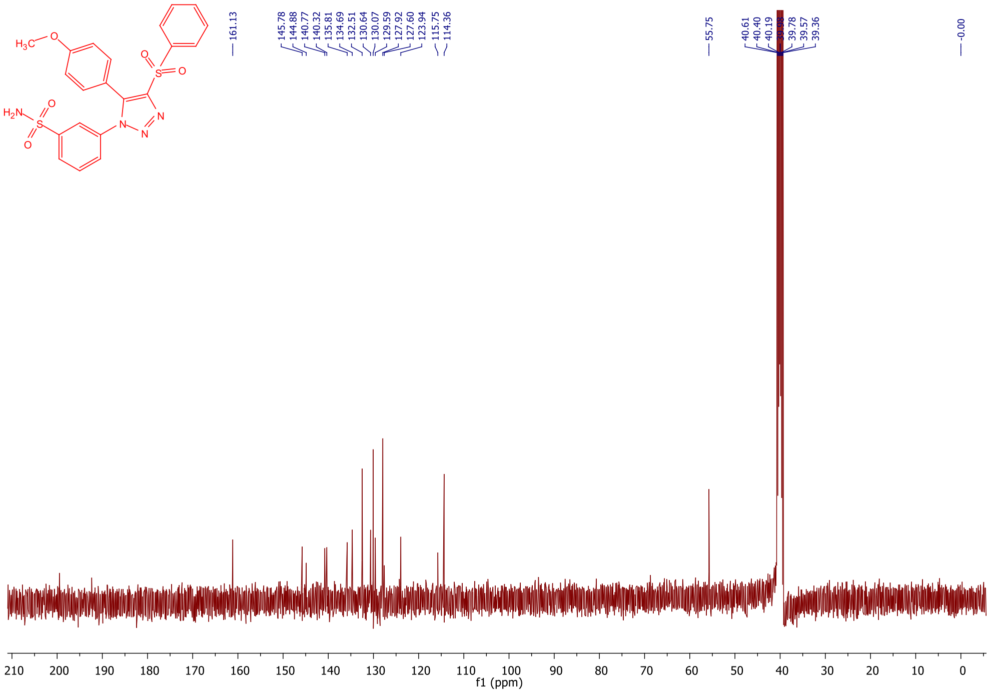

(10h) ^{1}H NMR

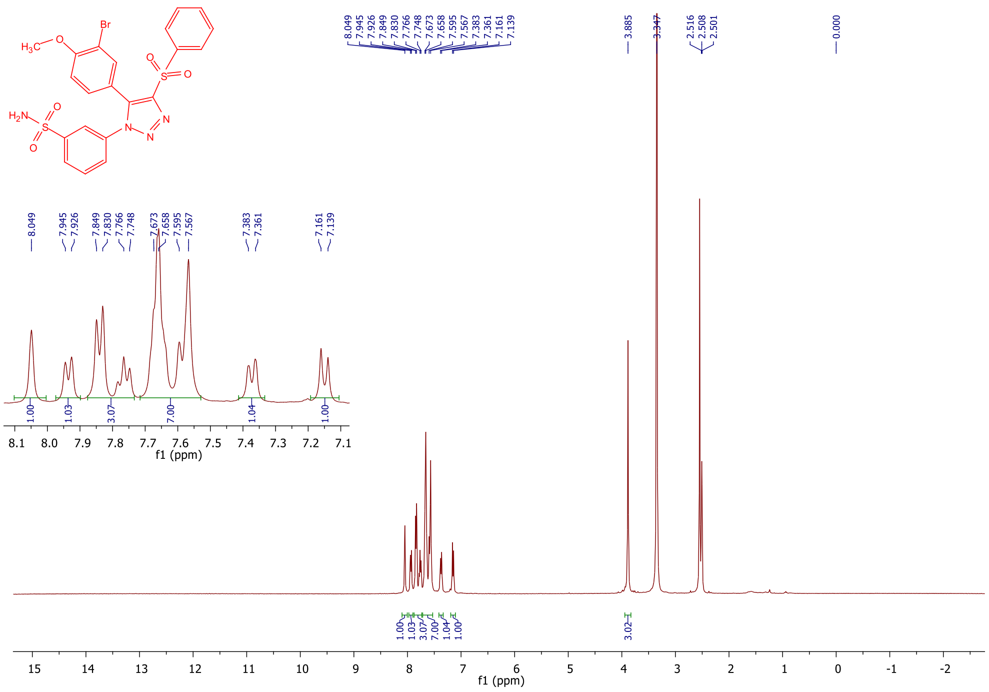

(10h) ^{13}C NMR

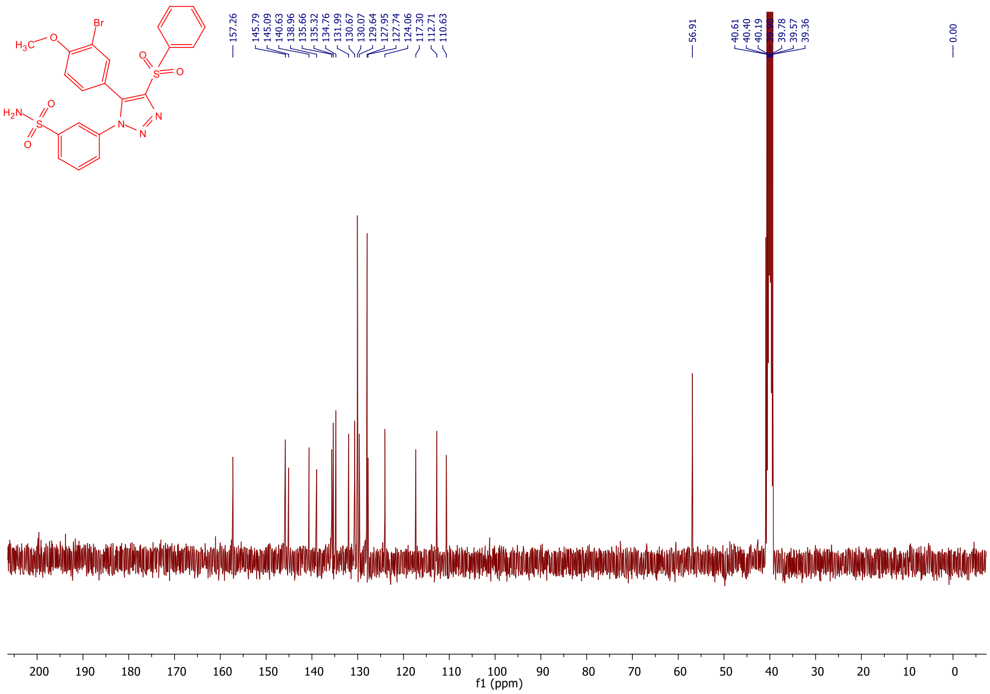

(10i) ^{1}H NMR

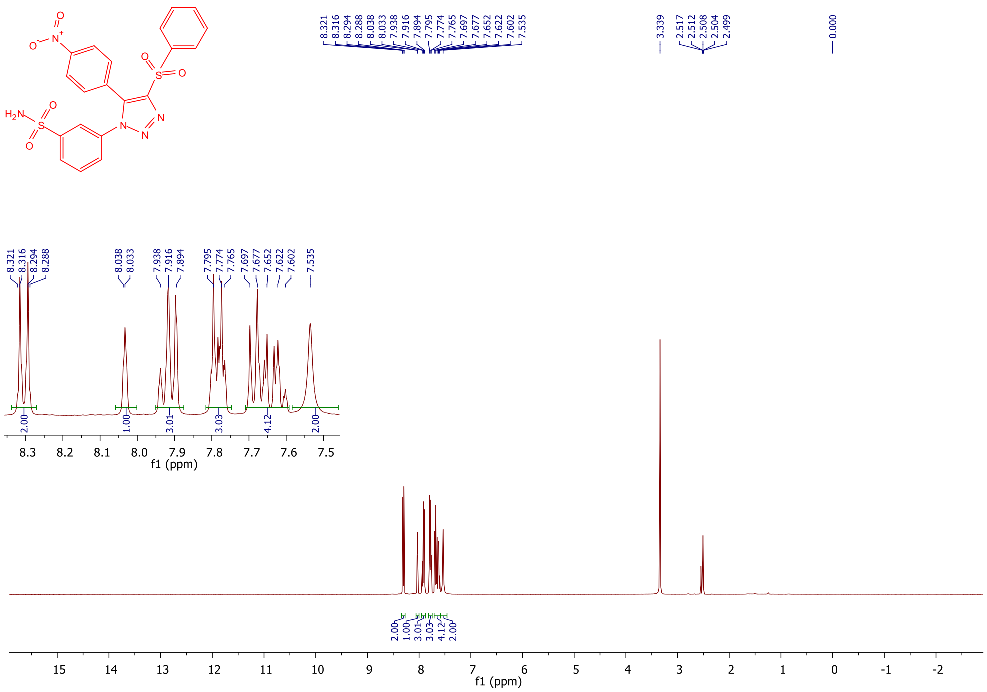

(10i) ^{13}C NMR

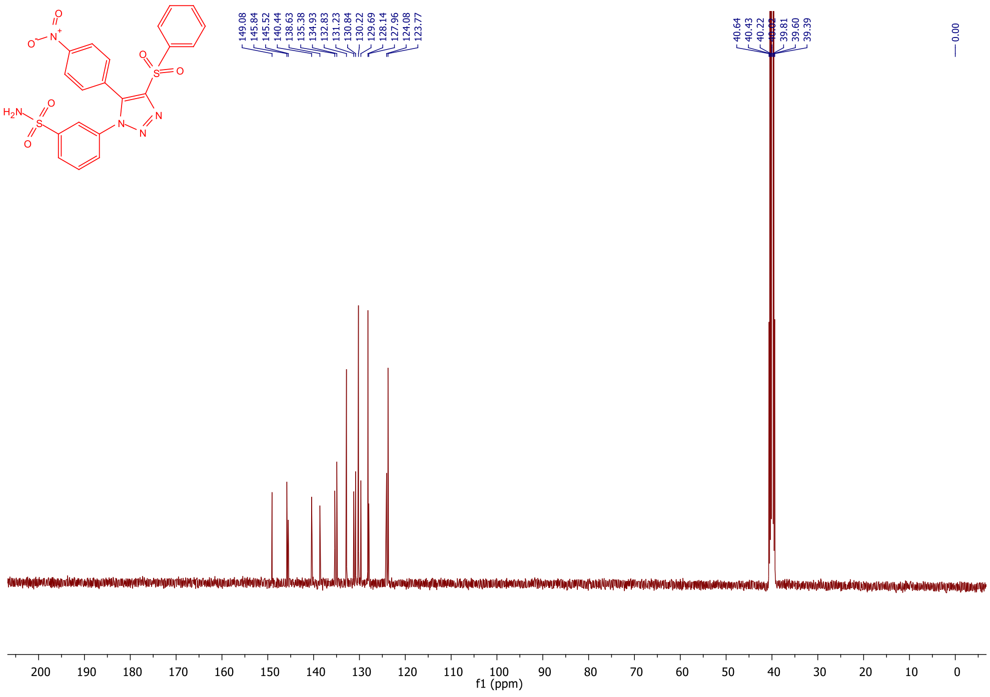

(10j) ^{1}H NMR

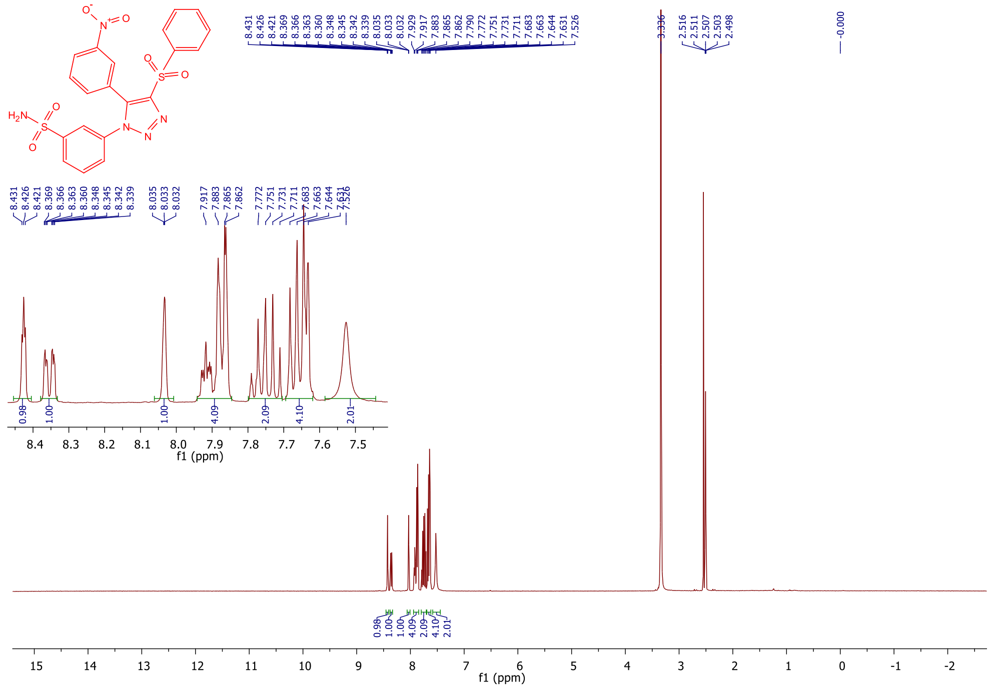

(10j) ^{13}C NMR

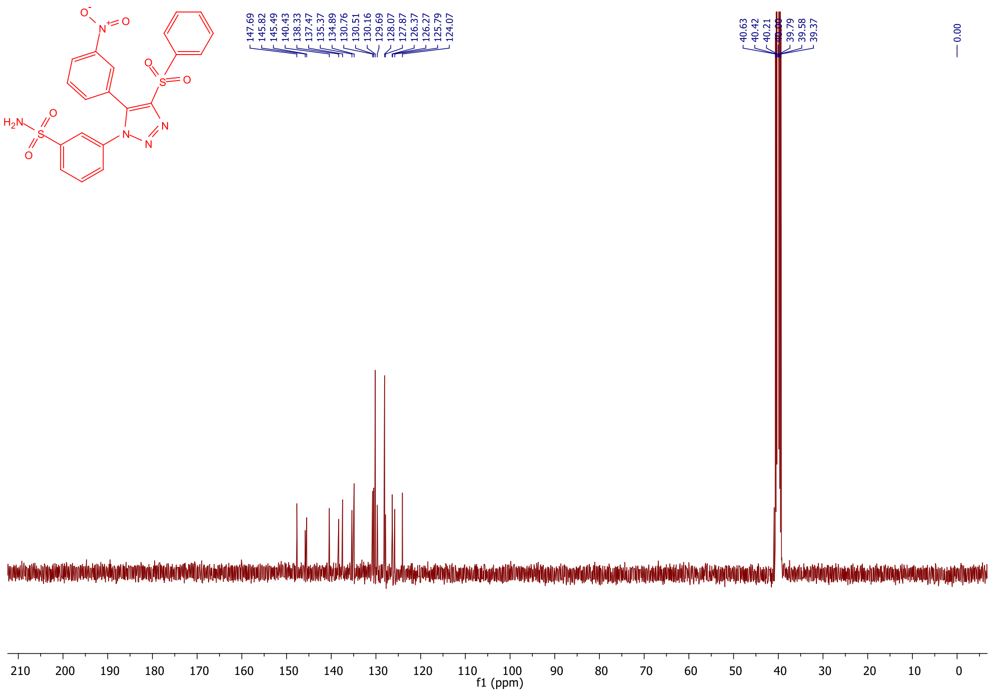

(10k) ^{1}H NMR

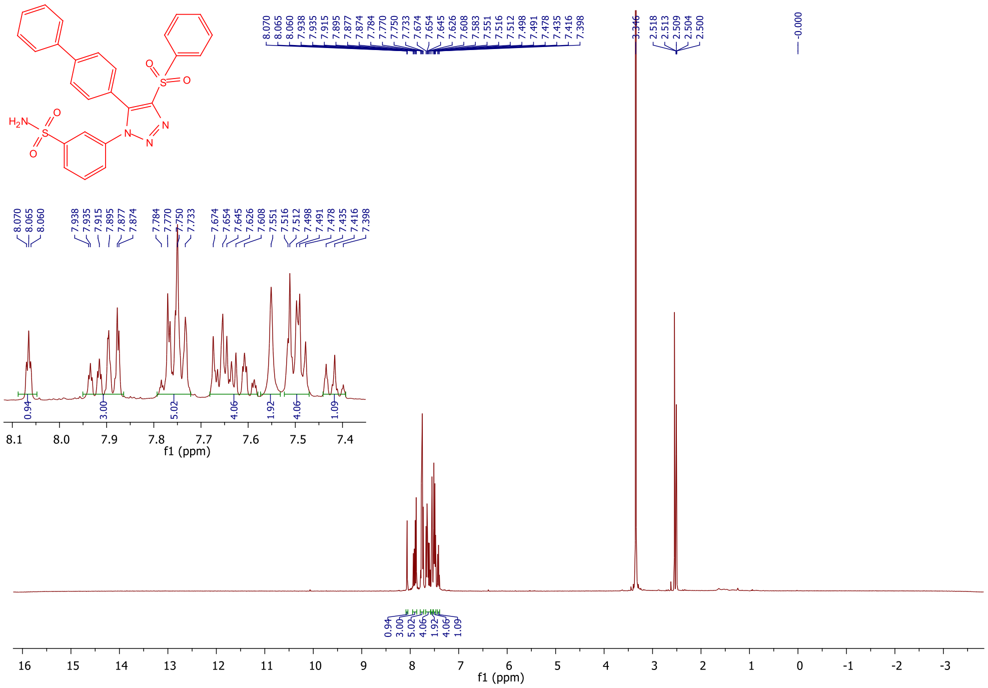

(10k) ^{13}C NMR

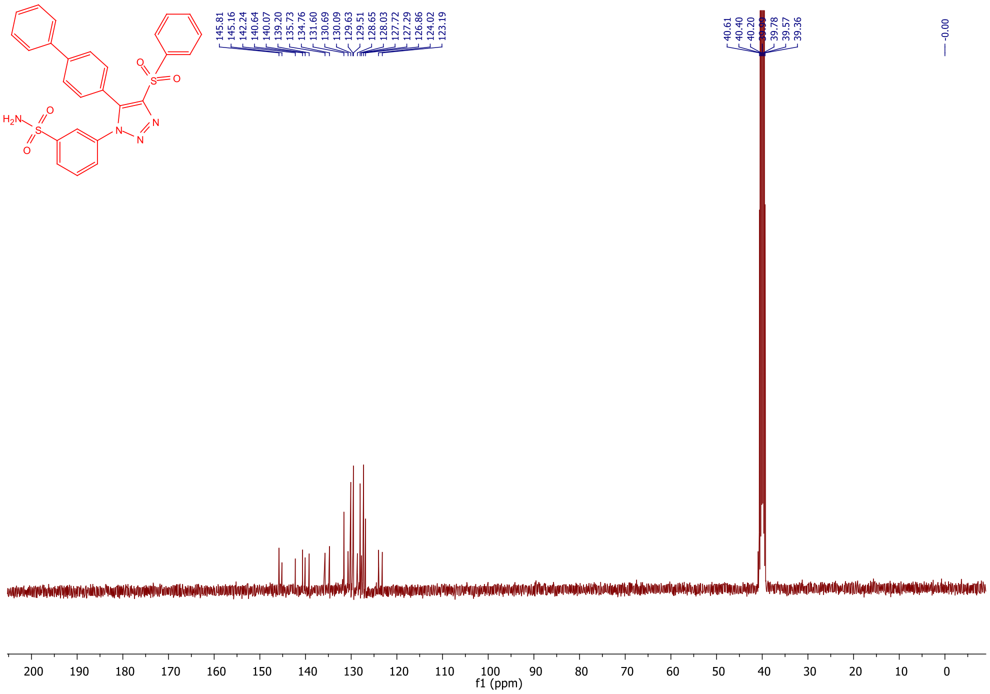

Supplement: Supplemental Material [file IENZ_A_2077333_SM6685.pdf]
